# Scoring and validation of a simple model for predicting diabetic retinopathy in patients with type 2 diabetes based on a meta-analysis approach of 21 cohorts

**DOI:** 10.1080/07853890.2024.2413920

**Published:** 2024-10-11

**Authors:** Hang Guo, Fei Han, Jing-Ru Qu, Cong-qing Pan, Bei Sun, Li-Ming Chen

**Affiliations:** NHC Key Laboratory of Hormones and Development, Tianjin Key Laboratory of Metabolic Diseases, Chu Hsien-I Memorial Hospital and Tianjin Institute of Endocrinology, Tianjin Medical University, Tianjin, China

**Keywords:** Diabetic retinopathy, clinical prediction model, meta-analysis, cohort study

## Abstract

**Aim:**

To develop and validate a model for predicting diabetic retinopathy (DR) in patients with type 2 diabetes.

**Methods:**

All risk factors with statistical significance in the DR prediction model were scored by their weights. Model performance was evaluated by the area under the receiver operating characteristic (ROC) curve, Kaplan–Meier curve, calibration curve and decision curve analysis. The prediction model was externally validated using a validation cohort from a Chinese hospital.

**Results:**

In this meta-analysis, 21 cohorts involving 184,737 patients with type 2 diabetes were examined. Sex, smoking, diabetes mellitus (DM) duration, albuminuria, glycated haemoglobin (HbA1c), systolic blood pressure (SBP) and TG were identified to be statistically significant. Thus, they were all included in the model and scored according to their weights (maximum score: 35.0). The model was validated using an external cohort with median follow-up time of 32 months. At a critical value of 16.0, the AUC value, sensitivity and specificity of the validation cohort are 0.772 ((95% confidence interval (95%CI): 0.740–0.803), *p* < .01), 0.715 and 0.775, respectively. The calibration curve lied close to the ideal diagonal line. Furthermore, the decision curve analysis demonstrated that the model had notably higher net benefits. The external validation results proved the reliability of the risk prediction model.

**Conclusions:**

The simple DR prediction model developed has good overall calibration and discrimination performance. It can be used as a simple tool to detect patients at high risk of DR.

## 1. Introduction

With an increasing incidence rate, diabetes has become one of the major diseases that seriously threaten human health. The complications of diabetes involve multiple organs of the body. For instance, diabetic retinopathy (DR) is one of the common severe complications [1]. In 2019, the American Academy of Ophthalmology (AAO) reported a DR prevalence of about 34.6% and a vision-threatening DR prevalence of 10.2% in global diabetes patients [2, 3]. DR is the leading cause of blindness among the working-age population in developed countries, greatly impairing the quality of life of patients [4]. On account of the insidious symptoms of early DR, patients often lack subjective symptoms. Besides, there is currently no effective clinical treatment to save the vision of patients with proliferative DR. In addition, the duration of diabetes and hyperglycaemia have been shown to be important risk factors for the development and progression of DR [5]. However, many long-term diabetes patients do not have DR, even though they may have multiple risk factors. Therefore, effective DR management requires an in-depth investigation of DR susceptibility factors, early detection, prevention and treatment. Early and accurate identification of patients at risk of DR is the prerequisite to effective intervention, and it can help curb the progression of DR and lower the risk of blindness.

At present, fundoscopy is the main tool for DR screening, but it cannot predict the risk of DR in patients at a specified time point. In addition, the vast majority of currently available DR risk prediction models are constructed using population-based data and have been validated in many different countries, but there is still a lack of sufficient evidence to support them. Besides, their performance needs to be further improved. Type 2 diabetes coexists with multiple metabolic disorders. Unhealthy lifestyle and other factors are related to the pathogenesis of DR. A comprehensive assessment of these risk factors, early detection and individualized intervention for high-risk patients may be the most effective strategy to prevent DR. The purpose of this paper is to develop and validate a stable, reliable and easy-to-apply risk prediction model for DR in clinical practice and public health, and determine its value of predicting DR.

## 2. Method

### 2.1. Systematic review registration

This meta-analysis was registered in the International Prospective Register of Systematic Reviews (PROSPERO) (registration number: CRD42022350210).

### 2.2. Study population

#### 2.2.1. Derivation cohort

**Figure 1.**
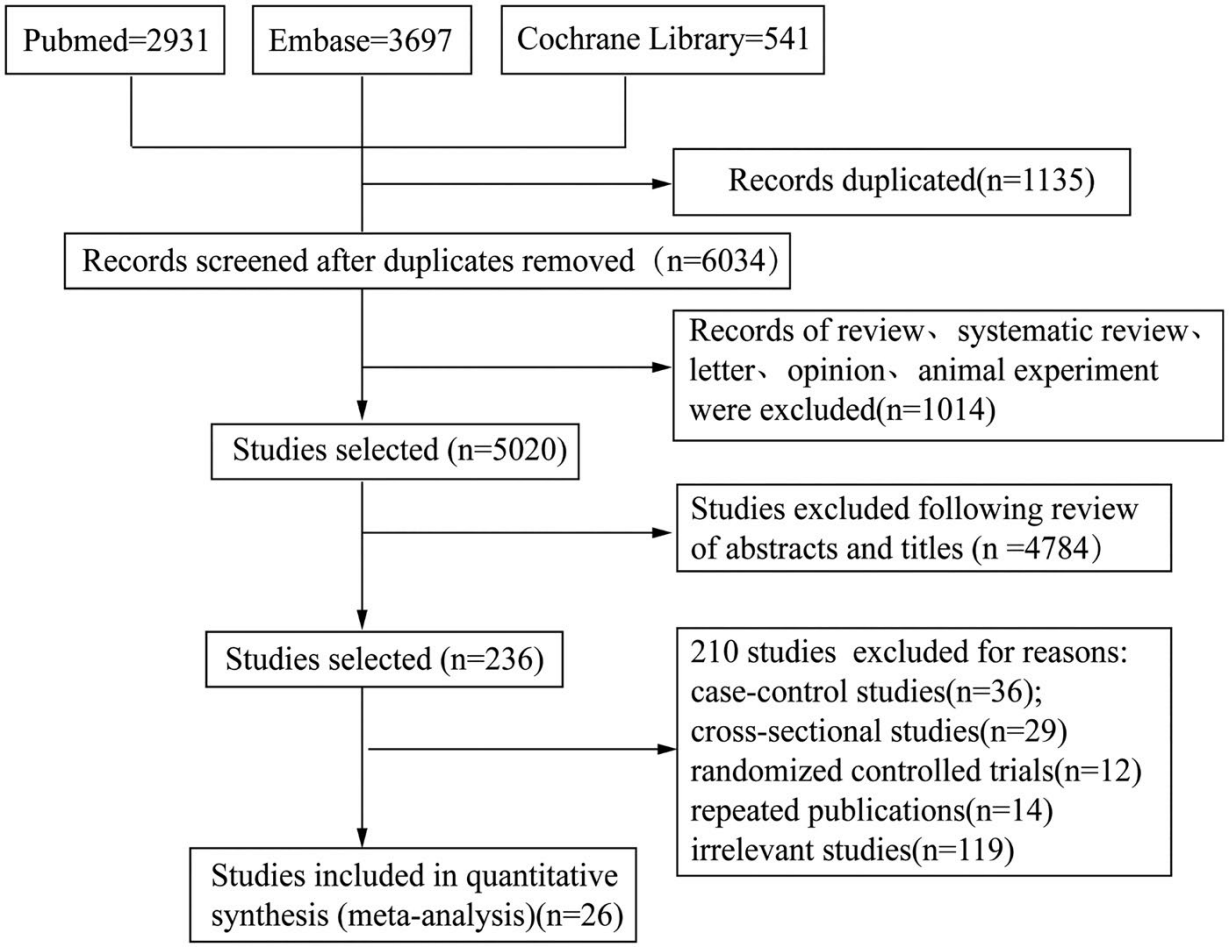
Flow chart of literature search and study selection for risk factors of DR development in patients with type 2 diabetes.

The derivation cohort was acquired through a systematic review and meta-analysis of 21 cohort studies [6–26], of which 18 studies were prospective and three studies were retrospective. Papers were retrieved from such electronic databases as PubMed, Cochrane Library and Embase from their inception to July 2022 by adopting the MeSH heading search strategy or searching keywords including ‘diabetes’, ‘retinopathy’, ‘risk factors’, ‘prospective studies’ and ‘cohort studies’. Finally, a total of 184,737 type 2 diabetic patients were enrolled in the derivation cohort, and they came from Asia (China, Bangladesh, Japan, Singapore, Taiwan, South Korea, Lebanon and Iran), Europe (France, Germany, Italy and Spain), America (United States and Canada) and Africa (Malawi). All included cohort studies reported risk ratios (RRs) and corresponding 95% confidence interval (95%CI) of risk factors, and were assessed using the Newcastle-Ottawa Scale (NOS) [27]. The flowchart of the literature screening method is shown in Figure 1. The specific search strategy, inclusion criteria, data extraction and quality assessment are summarized in Supplementary Data.

#### 2.2.2. Validation cohort

**Figure 2.**
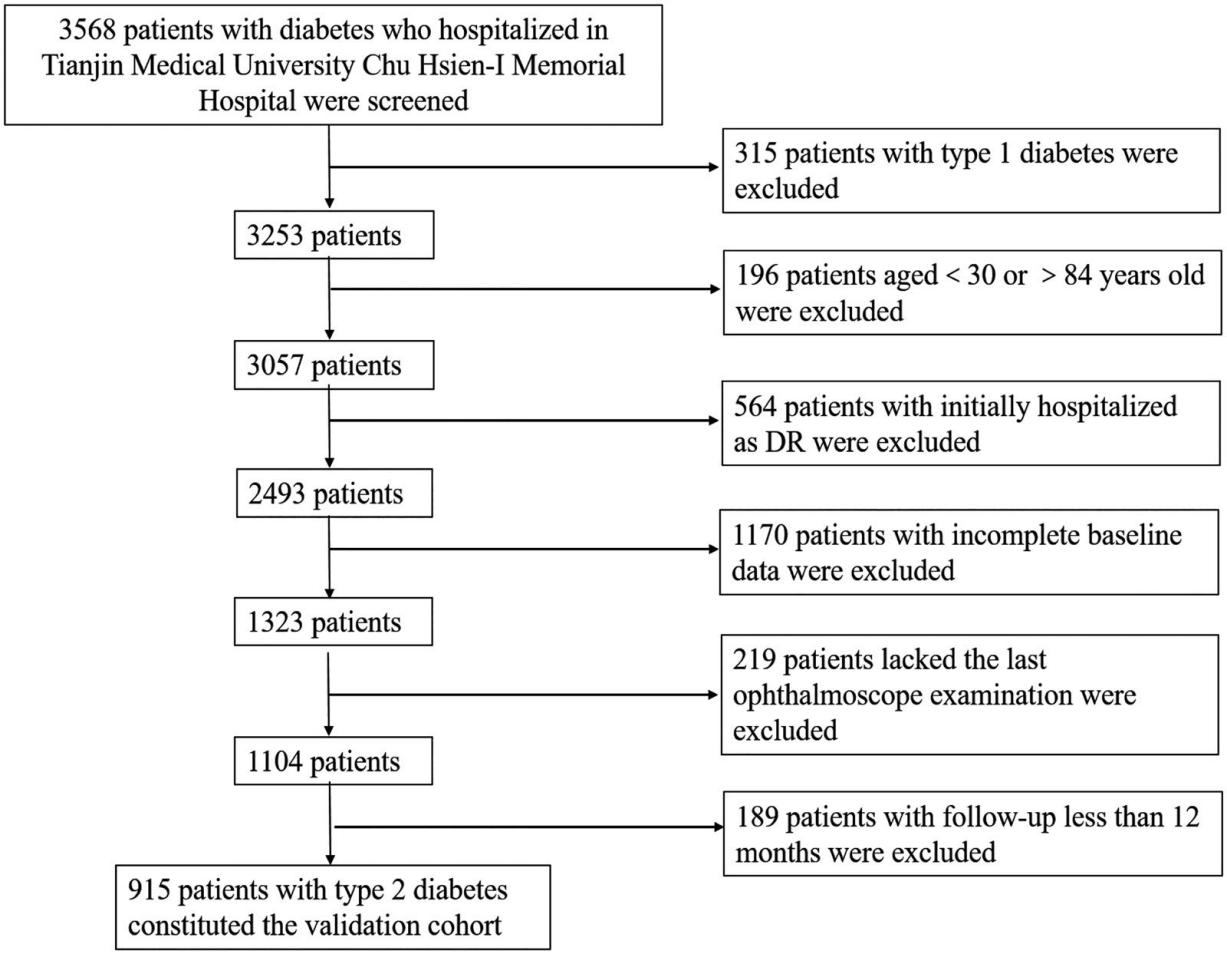
Process for the selection of patients with type 2 diabetes in the validation cohort.

We conducted a retrospective cohort study using the diabetes-specific database from the Big-data Intelligence Platform of Tianjin Medical University Chu Hsien-I Memorial Hospital (Metabolic Disease Hospital). To select the patients for the validation cohort, a retrospective cohort study of type 2 diabetes patients from Tianjin Medical University Chu Hsien-I Memorial Hospital (Metabolic Disease Hospital) was conducted (baseline from June 2016 to June 2021). To be included in the validation cohort, patients needed to be between 30 and 84 years old. These patients did not develop DR at baseline, and be with a follow-up for 1–5 years. Patients hospitalized for acute eye injury, malignancy, myocardial infarction, end-stage renal disease, acute infection or other reasons during the follow-up period were excluded. In the inclusion process, 3568 patients with diabetes who hospitalized in Tianjin Medical University Chu Hsien-I Memorial Hospital were screened at first. Then, 315 patients with type 1 diabetes, 196 patients aged below 30 or above 84, 564 patients with DR at baseline, 1170 patients with incomplete baseline information, 219 patients without taking the last funduscopy and 189 patients followed up for less than 12 months were eliminated. Finally, 915 patients with type 2 diabetes were incorporated into the validation cohort (Figure 2).

### 2.3. Outcome

The outcome was the occurrence of retinopathy caused by diabetes. Clinicians performed relevant examinations according to the Diabetic Retinopathy Preferred Practice Pattern (PPP)-2019 guideline [28], and then made a diagnosis based on the examination results as per the DR diagnostic criteria in the guideline.

### 2.4. Model development

(1) All risk factors in the prediction model were acquired through the above systematic review and meta-analysis. Appropriate RRs and 95%CIs were selected by subgroup analysis or sensitivity analysis, and then corresponding beta coefficients were calculated. (2) The beta coefficient is multiplied by 10, and then rounded to one decimal place (the last digit is 0 or 5) as the score of risk factor [29, 30]. (3) According to meta-analyses and clinical practice guidelines, all risk factors in the prediction model were classified, and their scores were computed. Finally, a risk scoring system was established. The scores of all risk factors were summed to obtain a total score [31]. A larger cumulative score indicated higher DR risk.

### 2.5. Model validation

External data from the aforementioned retrospective cohort studies were used to evaluate the risk prediction model constructed in this paper. The total scores of baseline variables in the validation cohort were calculated using the risk scoring system. The scores obtained were then used to draw receiver operating characteristic (ROC) curves, based on which the sensitivity, specificity, optimal cutoff and area under the ROC curve (AUC) were calculated. AUC values suggest the predictive performance of the model, with a numerical range of 0.5–1.0. A larger AUC value implies better prediction accuracy. According to the optimal cut-off point, patients were divided into four risk groups, namely low risk, low-intermediate risk, high-intermediate risk and high risk. To verify the performance of the prediction model, the incidence of DR was calculated, and the Kaplan–Meier curve was used to calculate the cumulative risk of different groups. Clinical validity or usefulness and the calibration degree were assessed by clinical decision and calibration curves, respectively. Statistical analysis was performed using SPSS 26.0 (IBM Corp., Armonk, NY) and STATA software version 15.1 (StataCorp, College Station, TX).

### 2.6. Ethics statement

This study was approved by the Institutional Review Board of Tianjin Medical University Chu Hsien-I Memorial Hospital and Tianjin Institute of Endocrinology. It was agreed to waive the requirement for informed consent, as this study was designed to retrospectively collect available data from articles published in peer-reviewed journals and databases

### 2.7. Statistical analysis

The 95%CI for the RR of each risk factor in the DR prediction model was extracted. Heterogeneity was quantified by the *Q* and *I*^2^ statistic tests. The fixed effect model would be adopted if the heterogeneity was not significant (*p* > .1, *I*^2^ < 50%). Otherwise, a random effects model would be applied. If there was heterogeneity, subgroup analysis or sensitivity analysis would be made to screen for heterogeneity depending on the type of study. Subgroup analyses were performed according to the magnitude of change in continuous variables and the classification of categorical variables. Continuous variables included diabetes duration (1-year increment), glycated haemoglobin (HbA1c) (increment by 1%), systolic blood pressure (SBP) (increment by 10 mmHg) and TG (increment by 1 mmol/L). The categorical variable was urinary protein (microalbuminuria and macroalbuminuria). RevMan (version 5.3.3; The Cochrane Collaboration, London, UK) and STATA software (version 15.1 StataCorp, College Station, TX) were employed for statistical analysis of the data. Unless otherwise specified, *p* values less than .05 were considered statistically significant.

## 3. Results

### 3.1. Derivation cohort

The baseline data of the participants of the cohort study were analysed, and the results suggested that a total of 184,737 diabetic patients aged 30–84 were recruited in the derivation cohort. About 52.8% of the enrolled patients were male, and their diabetes duration was 2–30 years. The follow-up period ranged from 2 to 17 years, equalling to 369,474–3,140,529 person-years. During the follow-up period, 34,324 patients developed DR, with an incidence rate of approximately 18.6%. In the cohort, 12–47% of patients received insulin therapy, 57–75% took oral antidiabetic drugs (OADs) and 20.6–43.2% were treated by lipid-lowering therapy. The ranges of mean HbA1c, mean SBP and mean TG were 7.3–12.0% (56.3–108 mmol/mol), 120–150 mmHg and 1.50–2.55 mmol/L, respectively. The baseline characteristics of the derivation cohort are shown in Supplementary Table 1. The derivation cohort contained 21 studies, which were evaluated as high quality by the Newcastle-Ottawa Quality Assessment Scale (provided in Supplementary Table 2). These cohort studies reported 13 risk factors, including age, sex, smoking, hypertension, diabetes mellitus (DM) duration, albuminuria, BMI, HbA1c, FBG, SBP, LDL, TC and TG (provided in Supplementary Table 3). The publication bias of all DR risk factors is shown in Supplementary Table 4.

### 3.2. Validation cohort

The validation cohort engaged a total of 915 Chinese patients with type 2 diabetes, including 574 males (62.7%) and 341 females (37.3%). The mean age ± SD was 53.6 ± 11.4 years, and the mean follow-up time was 32.0 months (interquartile range (IQR) = 22.0–40.0). At the end of follow-up, 330 patients (225 men and 105 women) progressed to DR. At baseline, the median duration of diabetes was 8.0 years [IQR = 3.0–13.0], the median SBP was 130.0 mmHg [IQR = 120.0–140.0], the median HbA1c level was 8.4% [68.0 mmol/mol], IQR = 7.2[55.0]–9.8[84.0], and the median triglyceride level was 1.74 mmol/L [IQR = 1.24–2.69]. Of the 915 cases, 400 patients (43.7%) were smokers, 254 patients (27.8%) had proteinuria (221 with microalbuminuria and 33 with macroalbuminuria), 488 patients (53.3%) received insulin therapy and 872 patients (95.3%) tool OADs. Basic information of patients in the validation cohort is introduced in Supplementary Table 5.

### 3.3. Model derivation

The 13 risk factors identified in the above meta-analysis were employed in the DR risk assessment model. Some subgroup or sensitivity analysis results that covered heterogeneity and clinical availability were extracted. The risk factors used in the model were male (RR = 1.29; 95%CI: 1.17–1.43; *p* < .01), smoking (RR = 1.53; 95%CI: 1.07–2.19; *p* = .02), 1-year increase in DM duration (RR = 1.04; 95%CI: 1.03–1.05; *p* < .01), microalbuminuria (RR = 1.17; 95%CI: 1.12–1.21; *p* < .01), macroalbuminuria (RR = 1.45; 95%CI: 1.14–1.83; *p* < .01), 1% increase in the HbA1c level (RR = 1.25; 95%CI: 1.22–1.29; *p* < .01), 10 mmHg increase in SBP (RR = 1.16, 95%CI: 1.06–1.26; *p* < .01) and 1 mmol/L increase in the TG level (RR = 1.49; 95%CI:1.00–2.23; *p* = .05). Supplementary Figure 1 shows a forest plot of all these risk factors. Supplementary Figure 2 summarizes subgroup and sensitivity analyses. RR (95%CI) (Supplementary Figures 3–9), *β*-coefficients and scores of risk factors included in the DR risk prediction model are listed in Table 1. In summary, the simple DR risk prediction model developed achieved following results: sex (female = 0, male = 2.5), smoking (no = 0, yes = 4.5), DM duration (years; <5.0 = 0, 5.0–9.9 = 2.5, 10.0–14.9 = 5, 15.0–19.9 = 7.5, ≥20 = 10), albuminuria (no = 0, microalbuminuria = 1.5, macroalbuminuria = 3.5), HbA1c (7.0% [53 mmol/mol] = 0, 7.0–7.9% [53–63 mmol/mol] = 2, 8.0–8.9% [64–74 mmol/mol] = 4, ≥9.0% [≥75 mmol/mol] = 6), SBP (mmHg; <130 = 0, 130–139 = 1.5, 140–149 = 3.0, ≥150 = 4.5) and TG (mmol/L; <1.70 = 0, ≥1.70 = 4.0). The model has a maximum score of 33.0 and is recommended for 30–84-year-old type 2 diabetes patients who have not progressed to DR (Table 2).

**Figure 3.**
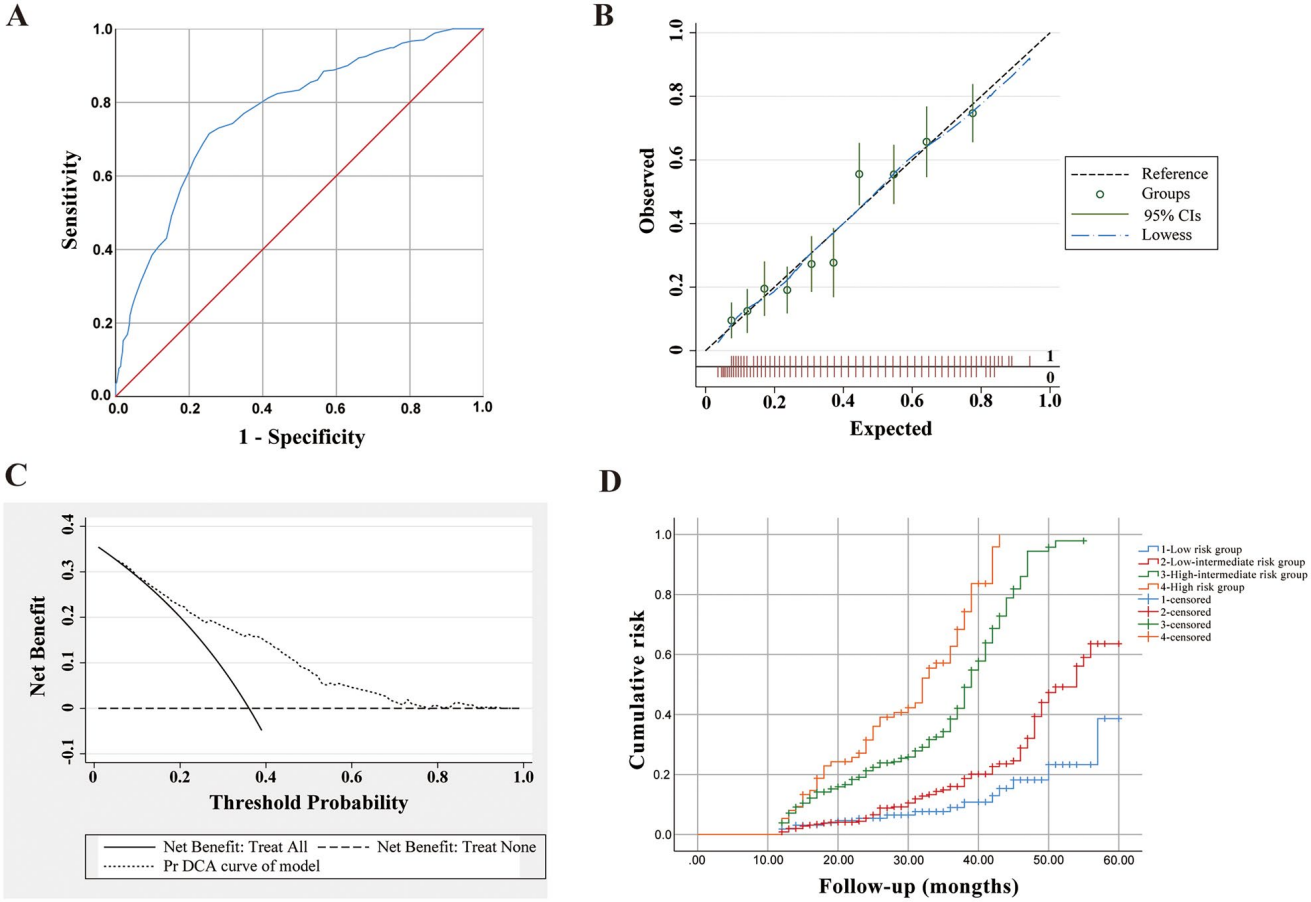
The performance of the DR risk prediction model in the validation cohort. (A) ROC curve for the DR risk prediction model. The AUC was 0.772 (95%CI 0.740–0.803). (B) Calibration curve of model in the validation cohort. (C) Net benefit curves of model for validation cohort. (D) Kaplan–Meier’s curve of DR endpoint for each risk group. Compared with the low risk group, low-intermediate risk group: RR = 2.21, 95%CI [1.33–3.69], *p* = .002, high-intermediate risk group: RR = 11.02,95%CI [6.69–18.14], *p* < .01, high risk group: RR = 29.04,95%CI [13.88–60.77], *p* < .01, low < 8.5, low-intermediate 8.5–16.0, high-intermediate 16.5–24.0, high 24.5–35.0.

**Table 1.** RR (95%CI), *β*-coefficients and scores of risk factors included in the DR risk prediction model.

Risk factors for DR	RR (95%CI)	*β*-coefficient	Scores
Sex (males)	1.29 [1.17, 1.43]	0.25	2.5
Smoking	1.53 [1.07, 2.19]	0.43	4.5
DM duration (by 1 year)	1.04 [1.03, 1.05]	0.04	0.5
Albuminuria			
Microalbuminuria	1.17 [1.12, 1.21]	0.16	1.5
Macroalbuminuria	1.45 [1.14, 1.83]	0.37	3.5
HbA1c (by 1% [11 mmol/mol])	1.25 [1.22, 1.29]	0.22	2.0
SBP (by 10 mmHg)	1.16 [1.06, 1.26]	0.15	1.5
TG (by 1 mmol/L)	1.49 [1.00, 2.23]	0.40	4.0

**Table 2.** Risk score model of DR prediction.

Risk factors for DR^a^	Category	Point
Sex	Female	0
	Male	2.5
Smoker^b^	No	0
	Yes	4.5
DM duration (years)	<5.0	0
	5.0–9.9	2.5
	10.0–14.9	5.0
	15.0–19.9	7.5
	≥20.0	10
Albuminuria	No	0
	Microalbuminuria	1.5
	Macroalbuminuria	3.5
HbA1c (% (mmol/mol))	<7.0 [<53]	0
	7.0–7.9 [53–63]	2
	8.0–8.9 [64–74]	4
	≥9.0 [≥75]	6
SBP (mmHg)	<130	0
	130–139	1.5
	140–149	3.0
	≥150	4.5
TG (mmol/L)	<1.70	0
	≥1.70	4.0

^a^ DR risk prediction model applied to patients with type 2 diabetes without DR.

^b^ Smoker was defined as having smoked more than 100 cigarettes in their lifetime. A highest score of risk assessment model is 35.0.

### 3.4. Model validation

According to the ROC curve of the DR risk prediction model in Figure 3(A), the AUC value of the validation cohort is 0.772 ((95%CI: 0.740–0.803), *p* < .01). The sensitivity, specificity and Youden index of different critical risk scores are shown in Supplementary Table 6. We used the maximum Youden index, which is 16.0, as the optimal critical risk score, and the corresponding sensitivity and specificity are 71.5% and 77.5%, respectively. In Figure 3(B), the deviation correction curve of the validation cohort is roughly consistent with the ideal curve on the whole scale, indicating that the model fits well and has a good calibration effect. This study conducted DCA to evaluate clinical efficacy and quantified the net benefit probability within a threshold of 0.0–1.0. There is a direct correlation between the decision curve and the net benefit of the model’s clinical decision. This correlation depends on the distance between the two extreme curves, with the model’s clinical decision being more advantageous at greater distances from these curves. In Figure 3(C), the DCA curve shows that when Pt is within 0.1–0.95, the model has higher clinical benefits. It demonstrates good clinical application value of the model in said range. According to the optimal cut-off point, 915 patients with type 2 diabetes were divided into four groups: low risk (*n* = 165), low-intermediate risk (*n* = 365), high-intermediate risk (*n* = 310) and high risk (*n* = 75). The risk scores of the above four groups were <8.5, 8.5–16.0, 16.5–24.0 and 24.5–35.0, respectively. It can be seen from Kaplan–Meier’s curves for DR groups (Figure 3(D)) that the RRs of developing DR in the low-intermediate, high-intermediate and high risk groups ((2.33, 95%CI: 1.33–4.08), (12.05, 95%CI: 6.95–20.90), (25.66, 95%CI: 12.45–52.97)) were higher than that in the low risk group (*p* < .05).

## 4. Discussion

DR is a complex multifactorial disease arising from genetic and environmental factors [32]. At present, in addition to controlling blood sugar and blood pressure (BP), some effective interventions can mitigate the progression of DR [33]. Therefore, an exploration of the risk factors of DR is vital to slowing the progression to DR. In this study, a simple and clinically applicable risk prediction model was developed through systematic reviews and meta-analyses. High-quality systematic reviews and meta-analyses are proven methods at the top of the evidence pyramid. Moreover, independent risk factors for the development of DR were identified from 21 high-quality cohort studies involving 184,737 individuals. The risk factors are sex, smoking, DM duration, albuminuria, HbA1c, SBP and TG levels. The model was validated using a cohort with median follow-up time of 32 months, and the results demonstrate the high reliability of the risk prediction model. All the risk factors can be artificially adjusted to delay the occurrence and decelerate the DR progress except for sex and the disease duration, which are uncontrollable. The model can reliably distinguish low-risk and high-risk patients. For low-risk patients, the interval of DR screening can be extended. Nevertheless, high-risk groups are recommended to take more intensive examinations and receive early intervention. Targeted interventions for patients at different risk levels can curb or even prevent the development of DR, thereby lightening the financial burden of individuals and governments.

In this study, smoking, TG and albuminuria were dominant risk factors in predicting the DR risk. The study results suggested that patients who smoked were more prone to develop DR (RR = 1.53, 95%CI: 1.07–2.19). This finding is confirmed by several other studies [34–36]. The reason may be that higher levels of carboxyhaemoglobin in smokers reduce the oxygen-carrying capacity of the blood, leading to retinal hypoxia and DR progression [37]. In addition, nicotine may cause vasoconstriction, increase oxidative stress and chronic inflammation, and thus aggravate DR [38, 39]. Therefore, quitting or reducing smoking is essential for diabetic patients to prevent DR. Dyslipidaemia, especially abnormally high TG levels, plays a crucial role in the development and progression of DR [40]. Chronic inflammation caused by T2DM leads to the accumulation of triglycerides in macrophages, thus aggravating DR [41]. In this study, it was found that the risk of DR increased by 49% with every 1 mmol/L increase of the TG level. Previous studies have shown a close relationship of DR with proteinuria [42] and diabetic nephropathy [43]. Most of the patients with diabetic nephropathy suffer from DR, so it is speculated that the two diseases have similar pathogenesis. It is generally believed that hyperglycaemia leads to metabolic abnormalities, enhances oxidative stress, and promotes cytokine release, consequently destroying the glomerular filtration membrane barrier and the blood–retinal barrier [44]. Thus, diabetic patients with high urinary protein levels should undergo the fundus examination as soon as possible to screen for DR. The results of this study revealed that both microalbuminuria and macroalbuminuria were associated with DR. The macroalbuminuria group had a higher risk of DR than the microalbuminuria group, suggesting that a higher albuminuria level relates to higher incidence of DR. Furthermore, the incidence of DR in male patients was higher than that in female patients, which is consistent with the findings of multiple studies [12, 15]. This may be due to genetic or lifestyle differences. In addition, HbA1c was also a risk factor. The risk of DR elevated by 25% with every 1% (11 mmol/mol) increase of the HbA1c level. Hence, keeping HbA1c at a reasonable level can tremendously reduce the risk of DR. According to the 2019 American Diabetes Association (ADA) guidelines [45], the HbA1c level of diabetic patients should be controlled below 6.5%, and those with higher HbA1c levels are more susceptible to complications. Similarly, some large prospective randomized controlled clinical studies have demonstrated the vital role of strict glycaemic control in DR prevention [46]. Elevated SBP has been claimed to be a risk factor for the development of DR [47], and an analysis of the United Kingdom Prospective Diabetes Study (UKPDS) [48] showed that strict control of BP could lower the risk of DR. Systolic BP was also determined as an important predictor of DR. An increase of 10 mmHg in the systolic BP level resulted in the rising of the DR risk by 16%. This may be attributed to the fact that the renin–angiotensin system in diabetic patients is activated by chronic hyperglycaemia. As a result, the level of angiotensin II (AII) in the vitreous body of DR patients ascends, leading to enhanced vascular permeability and angiogenesis [49, 50]. In addition, DR duration was identified as another major risk factor of DR, which is in good agreement with some findings. The duration of diabetes represents the period during which a diabetes patient is exposed to many risk factors. Therefore, longer disease duration contributes to higher incidence of various diabetes-related chronic complications. Some clinical guidelines and consensuses recommend immediate DR screening after the diagnosis of DR [2] because it can notably reduce the risk of vision loss in diabetic patients.

The current DR prediction models can predict the occurrence and progression of DR, but they have shortcomings. First, a vast majority of the available DR prediction models are developed based on small cross-sectional studies or post hoc analysis of case-control studies [51–53]. Second, researchers are subjective in the selection of risk factors, which leads to a big difference in the risk factors included in different models. Finally, the calculation methods applied in these models are complex and abstract, making it hard to apply them in clinical practice. Different from previous research, we conducted a systematic review and meta-analysis to build a DR risk assessment model based on a high-quality cohort study of 180,000 people. The clinical prediction model constructed is stable, with universality and high accuracy. It integrates data from multiple independent studies, showing enhanced statistical power and lower bias. In addition, the model we developed only includes seven common variables without complex mathematical calculations. Therefore, this model can be easily applied to the evaluation of the development of the disease at an early stage and thus helps the doctor to take timely intervention measures according to the corresponding risk factors.

In addition, 915 patients with type 2 diabetes from Tianjin Medical University Chu Hsien-I Memorial Hospital (Metabolic Disease Hospital) were recruited in the validation cohort to verify the predictive value of the model. The predictions fitted well and showed good calibration effects, and the DCA curve had higher net gains than the two reference lines. The above results indicate that the model is applicable to clinical practice. The AUC value was 0.772 (95%CI 0.740–0.803), and the maximum score of the model was 35.0. Taking 16.0 as the optimal cutoff value, the sensitivity and specificity were 71.5% and 77.5%, respectively. Based on their optimal cut-off point, the patients were further divided into low risk, low-intermediate risk, high-intermediate risk and high risk groups. Compared with low-risk patients, those at high-intermediate risk and high risk had 12.05-fold and 25.66-fold higher odds of developing DR, respectively. The model established can predict the risk of developing DR in patients with type 2 diabetes. The main goal of the model is to help doctors to develop reasonable intervention strategies for DR patients in the early stage according to their respective risk factors, and to enable patients to remain at or transform into low risk.

However, this study has some limitations. First, due to differences in the design, ethnic and sex compositions of the cohort studies, heterogeneity in the meta-analysis is inevitable. Although subgroup and sensitivity analyses are made to reduce heterogeneity in some studies, sources of heterogeneity for a few risk factors are not identified. Second, patients in our derivation cohort were from several countries, but we validated the prediction model with only retrospective cohorts from Chinese patients with diabetes. Therefore, further validation of the discriminating effect of the model necessitates studies of larger scale. Third, the lack of validation from multi-centre trials is a limitation of our study. We will include patients from more centres for further validation in the future. Last, our current prediction model can only predict whether a diabetic patient is currently at high risk of developing DR. It cannot be used to predict whether a diabetic patient will develop mild, moderate or severe DR.

## 5. Conclusions

A simple DR risk prediction model that integrates the lifestyle and clinical risk factors is developed based on a meta-analysis. The risk factors studied include sex, smoking, DM duration, albuminuria, HbA1c, SBP and TG. The predictive effect of the model is tested using an external validation cohort. The model is shown effective in early detection of DR and can facilitate the early intervention of DR. Therefore, the model can be widely used to guide early clinical decision-making in patients with type 2 diabetes.

## Supplementary Material

Supplementary_Files.docx

## Data Availability

The data that support the findings of this study are available on request from the corresponding author upon reasonable request.
