# Supplementary material for: Scoring and validation of a simple model for predicting diabetic retinopathy in patients with type 2 diabetes based on a meta-analysis approach of 21 cohorts
Source: Ann Med. 2024 Oct 11;56(1):2413920. doi: 10.1080/07853890.2024.2413920 (PMC11485693; doi:10.1080/07853890.2024.2413920)
Supplement: Supplementary_Files.docx [file IANN_A_2413920_SM6936.docx]

**SUPPLEMENTARY DATA**

**Systematic review and meta-analysis to identify the risk factors for diabetic retinopathy (DR) in patients with Type 2 diabetes.**

This study was based on the Preferred Reporting Items for Systematic and Meta-analysis (PRISMA) statement^[1]^ and the Meta-analysis of Observational Studies in Epidemiology (MOOSE) statement^[2]^.

**Search strategy**

Pubmed, Embase, and Cochrane Library were searched about the risk factors for DR from the time of their inception to July 2022 by the following medical subject heading (MeSH) terms or keywords: “diabetes”, “retinopathy”, “risk factors”, “prospective studies” and “cohort studies”. Two independent authors (Hang Guo and JingRu Qu) independently screened all titles and abstracts to determine. The full-texts were browsed when study could not be determined by the abstracts. Any disagreements were resolved through consulting two other senior researchers (Bei Sun and LiMing Chen) to attain consensus.

**Detailed search strategy in Pubmed：**

#1 " Diabetic retinopathy "[Mesh]

#2(((Diabetic Retinopathies[Title/Abstract]) OR (Retinopathies, Diabetic [Title/Abstract])) OR (Retinopathy, Diabetic[Title/Abstract]))

#3 #1 OR #2

#4 ((((((((((((((("Risk Factors"[Mesh]) OR "Sex"[Mesh]) OR "Body Mass Index"[Mesh]) OR "Obesity"[Mesh]) OR "Lipids"[Mesh]) OR "Triglycerides"[Mesh]) OR "Cholesterol"[Mesh]) OR ( "Cholesterol, HDL"[Mesh] OR "Cholesterol, LDL"[Mesh] )) OR "Albuminuria"[Mesh]) OR "Hyperlipidemias"[Mesh]) OR "Hypertension"[Mesh]) OR "Hyperglycemia"[Mesh]) OR "Hyperuricemia"[Mesh]) OR "Smoking"[Mesh]) OR "Glycated Hemoglobin A"[Mesh]) OR "Diabetic Nephropathies"[Mesh]

#5 ((((((age[Title/Abstract]) OR (diabetes duration[Title/Abstract])) OR (Diabetic kidney disease[Title/Abstract])) OR (Diabetic peripheral neuropathy[Title/Abstract])) OR (SBP[Title/Abstract])) OR (DPN[Title/Abstract])) OR (DBP[Title/Abstract])

#6 #4 OR #5

#7 (((("Cohort Studies"[Mesh]) OR "Follow-Up Studies"[Mesh]) OR "Longitudinal Studies"[Mesh]) OR "Prospective Studies"[Mesh]) OR "Retrospective Studies"[Mesh]

#8 #3 and #6 and #7

**Detailed search strategy in Embase：**

#1 'diabetic retinopathy'/exp

#2'diabetic retinopathies':ab,ti OR 'retinopathies, diabetic':ab,ti OR 'retinopathy, diabetic':ab,ti

#3 #1 OR #2

#4'risk factor'/exp OR 'sex'/exp OR 'body mass'/exp OR 'obesity'/exp OR 'lipid'/exp OR 'triacylglycerol'/exp OR 'cholesterol'/exp OR 'high density lipoprotein cholesterol'/exp OR 'low density lipoprotein cholesterol'/exp OR 'albuminuria'/exp OR 'hyperlipidemia'/exp OR 'smoking'/exp OR 'hemoglobin a1c'/exp OR 'diabetic nephropathy'/exp OR 'age'/exp OR 'diabetes duration'/exp OR 'diabetic neuropathy'/exp OR 'systolic blood pressure'/exp OR 'diastolic blood pressure'/exp

#5'hdl':ab,ti OR 'ldl':ab,ti OR 'diabetic kidney disease':ab,ti OR 'dkd':ab,ti OR 'sbp':ab,ti OR 'dpn':ab,ti OR 'dbp':ab,ti

#6 #4 OR #5

#7'cohort analysis'/exp OR 'longitudinal study'/exp OR 'prospective study'/exp OR 'retrospective study'/exp OR 'longitudinal'/exp

#8 #3 AND #6 AND #7

**Detailed search strategy in Cochrane Library：**

#1 MeSH descriptor: [Diabetic Retinopathy] explode all trees

#2 (diabetic retinopathies):ab,ti,kw or (retinopathies, diabetic):ab,ti,kw or (retinopathy, diabetic):ab,ti,kw

#3 #1 or #2

#4 MeSH descriptor: [Risk Factors] explode all trees

#5 (sex):ab,ti,kw or (Body Mass Index):ab,ti,kw or (obesity):ab,ti,kw or (lipid):ab,ti,kw or (triacylglycerol):ab,ti,kw or (cholesterol):ab,ti,kw or (high density lipoprotein cholesterol):ab,ti,kw or (low density lipoprotein cholesterol):ab,ti,kw or (albuminuria):ab,ti,kw or (hyperlipidemia):ab,ti,kw or (smoking):ab,ti,kw or (hemoglobin a1c):ab,ti,kw or (diabetic nephropathy):ab,ti,kw or (age):ab,ti,kw or (diabetes duration):ab,ti,kw or (diabetic neuropathy):ab,ti,kw or (systolic blood pressure):ab,ti,kw or (diastolic blood pressure):ab,ti,kw or (diabetic kidney disease):ab,ti,kw

#6 #4 or #5

#7 MeSH descriptor: [Cohort Studies] explode all trees

#8 (longitudinal studies):ab,ti,kw or (follow-up studies):ab,ti,kw or (prospective studies):ab,ti,kw or (retrospective studies):ab,ti,kw

#9 #7 or #8

#10 #3 and #6 and #9

**Inclusion and exclusion criteria**

Inclusion criteria: Studies were prospective or retrospective cohort studies based on original data; Data on the risk factors for DR reported as risk ratios (RRs) and 95% confidence intervals (95% CIs); Patients with type 2 diabetes without retinopathy at baseline will be eligible. Exclusion criteria: reviews, commentaries and editorials, irretrievable articles, animal studies and other studies from which data could not be extracted.

**Definition**

Type 2 diabetes was defined as fasting plasma glucose (FPG) of ≥ 7.0 mmol/L, 2-h plasma glucose (2-h PG) value of ≥11.1 mmol/L during a 75 g oral glucose tolerance test (OGTT), glycated hemoglobin A1c (HbA1c) of ≥6.5% (48mmol/mol), use of glucose-lowering drugs, self-reported diabetes, or using administrative data coding algorithms^[3, 4]^.DR was defined as development of non-proliferative DR (The ophthalmologist performed the dilated fundus examination (DFE) to evaluate and determine whether the patients with T2DM had progressed to DR.)

**Outcome**

Initiation of non-proliferative DR.

**Data extraction and quality assessment**

These meaningful data was extracted to **Supplementary Table 1** by two researchers (Hang Guo and JingRu Qu) independently, including first author, published year, country, details of the study design, sample size, age, DM duration, follow-up, risk factors and RR with 95% CI in each group. We assessed the quality of cohorts using the Newcastle-Ottawa scales. (The NOS contains eight entries and divides into three dimensions: selection, comparability, and otcome). The NOS score ranged from 0 to 9. Score of included articles should be 7 or more, The higher the score, the higher the quality of articles. (Shown in **Supplementary Table 2**)

**Statistical analysis**

The statistical analysis of the data was performed using RevMan (version 5.3.3; The Cochrane Collaboration) and STATA software, version 15.1 (StataCorp, College Station, TX). The results are presented as the risk ratios (RRs) with a 95% confidence interval (95%CI), and a *P*-value <0.05 was considered statistically significant unless otherwise specified. In addition, heterogeneity was quantified using the Q test and *I*^2^ statistics. When the heterogeneity test indicated no significant (*P*>0.1and I^2^<50%), a fixed-effects model was applied; otherwise, a random-effects model was used. Publication bias was assessed using Egger's and Begg's linear regression tests.

**Results**

We found 2931 articles from Pubmed, 541 from Corchrane Library, 3697 from Embase. 1135 duplicate articles were excluded. 1014 articles such as reviews and animal experiments were excluded. 4784 articles were excluded after carefully reviewing the titles and abstracts. Of the 236 articles that underwent full-text evaluation, 26 articles met our inclusion criteria^[5-30]^.

**Characteristics and quality of included studie**s

According to the Newcastle Ottawa scale, 5 studies had a score of 6, 2 studies had a score of 7, 7 studies had a score of 8, and 12 studies had a score of 9 (Shown in **Supplementary Table 2**). Included articles should have a score of 7 or more, so 21 articles were included in the study.

Of the 21 included studies, 18 were prospective cohorts and 3 were retrospective cohorts. There were 184737 patients with diabetes and 34324 incident DR cases were observed during follow-up, and the estimated incidence of DR in type 2 diabetic patients was 18.6%. The duration of follow-up ranged from 2 to 17 years, the age of the study participants was between 30 to 84. 5.7% of the participants were from Asia and Africa while 94.3% were from America and Europe. The characteristics of all these 21 cohorts were shown in **Supplementary Table 1**.

**Risk factors of DR in type 2 diabetic patients**

There were 13 risk factors available from the included cohorts, including age, sex, smoking, hypertension, diabetes mellitus (DM) duration, albuminuria, body mass index (BMI), hemoglobin A1c (HbA1c), fasting plasma glucose (FBG), systolic blood pressure (SBP), low density lipoprotein (LDL), total cholesterol (TC), triglyceride (TG), (Shown in **Supplementary Table 3**). Begg's and Egger's linear regression tests were used to examine publication bias. All risk factors did not show publication bias, and the overall publication bias was relatively good. The publication bias of all DR risk factors is shown in Supplementary Table 4.

Of the 7 risk factors for meta-analysis, there were sex, smoking, DM duration, albuminuria, HbA1c, SBP and TG with pooled RRs of 1.29,1.53,1.04,1.32,1.25,1.16 and 1.49, respectively. Considering the feasibility of clinical practice, we chose the results from subgroup analysis or sensitivity analysis which were more reasonable. Male (RR=1.29; 95%CI 1.17-1.43; *P*<0.01). Smoking (RR=1.53; 95%CI 1.07-2.19; *P*=0.02); DM duration increment by 1 year (RR=1.04; 95%CI 1.03-1.05; *P*<0.01); Microalbuminuria (RR=1.17; 95%CI 1.12-1.21; *P*<0.01); Macroalbuminuria (RR=1.45; 95%CI 1.14-1.83; *P*<0.01); HbA1c increment by 1% (RR=1.25; 95%CI 1.22-1.29; *P*<0.01); SBP increment by 10 mmHg (RR=1.16, 95% CI 1.06-1.26; *P*< 0.01); TG increment by 1 mmol/L (RR=1.49; 95%CI 1.00-2.23; *P*=0.05).The details of these risk factors in the involving studies are shown in **Supplementary Figure 3-9.**

**Supplementary Table 1.** **Baseline characteristics and risk factors of the 26 cohort studies**

| First author | Year | Country/Region | Study design | Sample size  (male/%) | Follow-up  (years) | Age  (years) | DM duration  (years) | Risk  factors |
| --- | --- | --- | --- | --- | --- | --- | --- | --- |
| Ahmed | 2012 | Bangladesh | retrospective  cohort | 468 (47.9) | 15 | 41 ± 8 | NR | 1,2,4,8,13,14,15 |
| Anwar | 2019 | America | prospective cohort | 57 (57) | 2 | 50.72±9.29 | 8.31±6.83 | 2,3,4,5 |
| Araki | 1993 | Japan | prospective cohort | 36(36) | 7.9 | 69.2 ± 5.5 | 6.9±7.9 | 1,2,4,5,6,7,8,  11 |
| Burgess | 2017 | Malawi | prospective cohort | - | 5.3 | 52 (45-58) | 3.9 (2.2-7.8) | 1,5,8,15 |
| Chamard | 2021 | France | prospective cohort | 54815(54.1) | 13 | 55 (47–62) | - | 8 |
| Cheung | 2021 | Singapore | prospective cohort | 251(50.4) | 8 | 63.2±8.9 | 9.4±7.9 | 1,2,8,9 |
| Chiu | 2021 | Taiwan | prospective cohort | 339(42.8) | 5 | 67.85±6.53 | - | 2,8,12 |
| Cikamatana | 2007 | Australia | prospective cohort | 1230(52.7) | 5 | 66.2±8.4 | 5.8±7.5 | 5,11 |
| Hammes | 2015 | Germany | prospective cohort | 33299(51.4) | 13 | 68.7±0.04 | 9.2±0.03 | 2,4, 6, 7,8 |
| Jones | 2012 | England | prospective cohort | - | 17 | - | - | 1 |
| Kumari | 2019 | Singapore | prospective cohort | 404 (53.23) | 6 | 59.24±9.4 | 7.09±7.78 | 1,2,3,4,5,6,7,  8,9 |
| Leske | 2003 | Africa | prospective cohort | 149(36.3) | 9 | 57.6±9.4 | 6.5±7.2 | 9 |
| Liu | 2015 | China | prospective cohort | 304 (48.98) | 5 | 69±5.87 | 5.04 ± 1.11 | 2,4,5,8,14 |
| Looker | 2003 | America | prospective cohort | 77(27.5) | 4.2 | 42.6 | 6.0 | 1,2,4,5,6,8,11,14 |
| Maberley | 2002 | Canada | retrospective cohort | 83(34.4) | 5 | 50.5 | 4.6 | 1,2,3,4,5,7,  10,13,14,15 |
| Manaviat | 2008 | Iran | prospective cohort | 31(25.8) | 4 | 55.2±9.64 | 11.6±6.2 | 2,3,5,7,9,10,  11,15, |
| Salinero-Fort | 2013 | Spain | prospective cohort | 1188(49.4) | 3 | 67.8±10.6 | 7.7±7.1 | 2,4,5,6,8,12 |
| Salti | 2009 | Lebanon | prospective cohort | 223(44.6) | 3 | 56.4±11.72 | 9.2±6.9 | 5,6,8,9 |
| Sardarinia | 2022 | Iran | prospective cohort | 494(42.3) | 12.7 | 55.4±11.5 | - | 1,2,3,7,11 |
| Semeraro | 2011 | Italy | prospective cohort | 3020(60) | 11 | 61.0±10.5 | 2.0 (0.0–8.0) | 2,5,8, 9 |
| Tseng | 2015 | Taiwan | retrospective cohort | 429(57.7) | 2.9 | - | - | 8,9,14 |
| Tudor | 1998 | America | prospective cohort | 74(43.6) | 5.4 | 58.1 | 4.6 | 1,2,3,5,7,9,10,13,14,15 |
| Tung | 2005 | Taiwan | prospective cohort | 301(41.5) | 2.56 | 56.2 ± 10.9 | - | 5,8,9,15 |
| Wang | 2022 | China | prospective cohort | 796(41.2) | 3 | - | - | 1,5,6,8,9,11 |
| Yoshida | 2001 | Japan | prospective cohort | 597(75.9) | 6.7 | 54 | 8 | 1,5,7,8,9,10,  14 |
| Yun | 2016 | South Korea | prospective cohort | 220(42.1) | 11.8 | 54.3±10.0 | 6.7±5.3 | 1,2,4,5,8,12 |

1.Age 2.Sex 3.Smoker 4. Hypertension 5.DM duration 6. Albuminuria 7.BMI 8.HbA1c 9.SBP 10.DBP 11.FBG 12.LDL 13.HDL 14.TC 15.TG

| **First author/year** | **Selection** | | | | **Compara-bility** | **Outcome** | | | **Total**  **scores** |
| --- | --- | --- | --- | --- | --- | --- | --- | --- | --- |
|  | Representa-tiveness of the exposed cohort | Selection of the non- exposed cohort | Ascertain-ment of exposure | Demonstration  that outcome of interest was not present at start of study | Comparability of cohorts on the basis of the design or analysis | Assess-  ment of outcome | Was follow-up long enough for outcomes to occur | Adequacy of follow up of cohorts |  |
| Ahmed 2012 | ☆ | ☆ | ☆ | ☆ | ☆☆ | ☆ | ☆ | - | 8 |
| Anwar 2019 | ☆ | ☆ | ☆ | ☆ | ☆☆ | - | - | ☆ | 7 |
| Araki 1993 | ☆ | ☆ | - | - | ☆ | ☆ | ☆ | ☆ | 6 |
| Burgess 2017 | ☆ | ☆ | ☆ | ☆ | ☆ | ☆ | ☆ | ☆ | 8 |
| Chamard 2021 | ☆ | ☆ | ☆ | ☆ | ☆☆ | ☆ | ☆ | ☆ | 9 |
| Cheung 2021 | ☆ | ☆ | ☆ | ☆ | ☆☆ | ☆ | ☆ | ☆ | 9 |
| Chiu 2021 | ☆ | - | ☆ | ☆ | ☆☆ | ☆ | ☆ | - | 7 |
| Cikamatana 2007 | ☆ | ☆ | - | - | ☆☆ | - | ☆ | ☆ | 6 |
| Hammes 2015 | ☆ | ☆ | - | ☆ | ☆☆ | ☆ | ☆ | ☆ | 8 |
| Jones 2012 | ☆ | ☆ | ☆ | - | ☆ | - | ☆ | ☆ | 6 |
| Kumari 2019 | ☆ | ☆ | ☆ | ☆ | ☆☆ | ☆ | ☆ | ☆ | 9 |
| Leske 2003 | ☆ | ☆ | ☆ | ☆ | ☆ | ☆ | ☆ | ☆ | 8 |
| Liu 2015 | ☆ | ☆ | ☆ | ☆ | ☆☆ | ☆ | ☆ | ☆ | 9 |
| Looker 2003 | ☆ | ☆ | ☆ | ☆ | ☆☆ | ☆ | ☆ | ☆ | 9 |
| Maberley 2002 | ☆ | ☆ | ☆ | ☆ | ☆☆ | ☆ | ☆ | - | 8 |
| Manaviat 2008 | ☆ | - | ☆ | - | ☆☆ | - | ☆ | ☆ | 6 |
| Salinero-Fort 2013 | ☆ | ☆ | ☆ | ☆ | ☆☆ | ☆ | ☆ | ☆ | 9 |
| Salti 2009 | ☆ | ☆ | ☆ | ☆ | ☆☆ | ☆ | ☆ | ☆ | 9 |
| Sardarinia 2022 | ☆ | ☆ | ☆ | ☆ | ☆☆ | ☆ | ☆ | ☆ | 9 |
| Semeraro 2011 | ☆ | ☆ | - | ☆ | ☆☆ | ☆ | ☆ | ☆ | 8 |
| Tseng 2015 | ☆ | ☆ | ☆ | ☆ | ☆☆ | ☆ | ☆ | ☆ | 9 |
| Tudor1998 | ☆ | ☆ | - | - | ☆ | ☆ | ☆ | ☆ | 6 |
| Tung 2005 | ☆ | ☆ | ☆ | ☆ | ☆☆ | ☆ | ☆ | ☆ | 9 |
| Wang 2022 | ☆ | ☆ | ☆ | ☆ | ☆☆ | ☆ | ☆ | ☆ | 9 |
| Yoshida 2001 | ☆ | ☆ | ☆ | ☆ | ☆☆ | ☆ | ☆ | ☆ | 9 |
| Yun 2016 | ☆ | ☆ | ☆ | ☆ | ☆☆ | - | ☆ | ☆ | 8 |

**Supplementary Table 2. Newcastle-Ottawa Quality Assessment Scale of the 26 cohort studies.**

**Supplementary Table 3**. **13 risk factors include in the systematic review and meta-analysis.**

| **Risk factors** | **First author/ Year** | **Sample size** | **No. of DR** | **Definition of risk factor** | **RR**  **(95% CI)** | **P value** |
| --- | --- | --- | --- | --- | --- | --- |
| Age | Ahmed 2012 | 977 | 494 | Increment by 1 year | 0.75 (0.61-0.92) | 0.006 |
| Age | Burgess 2017 | 135 | 40 | Increment by 1 year | 0.97 (0.92-1.01) | 0.094 |
| Age | Cheung 2021 | 498 | 96 | Increment by 1 year | 0.99 (0.97-1.02) | >0.05 |
| Age | Kumari 2019 | 759 | 93 | Increment by 1 year | 0.99 (0.96-1.01) | 0.299 |
| Age | Looker 2003 | 280 | 75 | Increment by 1 year | 1.01 (0.99–1.03) | >0.05 |
| Age | Sardarinia 2022 | 1169 | 187 | Increment by 1 year | 1.01 (0.99–1.04) | 0.31 |
| Age | Wang 2022 | 1932 | 856 | Increment by 1 year | 0.97 (0.95-0.98) | <0.05 |
| Age | Yoshida 2001 | 787 | 132 | Increment by 1 year | 1.01 (0.98–1.03) | 0.5672 |
| Age | Maberley 2002 | 241 | 83 | ≥60 (vs.<60) years | 1.38 (0.62–3.07) | >0.05 |
| Age | Yun 2016 | 523 | 235 | ≥50.5 (vs.<50.5) years | 1.40 (1.06–1.83) | 0.018 |
| Sex | Ahmed 2012 | 977 | 494 | Male | 0.91 (0.75-1.11) | 0.374 |
| Sex | Anwar 2019 | 100 | 9 | Male | 0.94 (0.24–3.72) | 0.927 |
| Sex | Cheung 2021 | 498 | 96 | Female | 1.05 (0.67-1.63) | >0.05 |
| Sex | Chiu 2021 | 792 | 611 | Female | 1.29(1.08-1.53) | 0.004 |
| Sex | Hammes 2015 | 64784 | 13034 | Male | 1.11 (1.07-1.15) | <0.0001 |
| Sex | Kumari 2019 | 759 | 93 | Male | 1.33 (0.87-2.02) | 0.184 |
| Sex | Liu 2015 | 622 | 52 | Male | 1.47(1.21-1.62) | <0.01 |
| Sex | Looker 2003 | 280 | 75 | Female | 0.88 (0.53–1.46) | >0.05 |
| Sex | Salinero-Fort 2013 | 2405 | 194 | Female | 1.12(0.84-1.49) | 0.451 |
| Sex | Sardarinia 2022 | 1169 | 187 | Male | 0.41 (0.21–0.79) | 0.01 |
| Sex | Semeraro 2011 | 5034 | 569 | Male | 1.31(1.05-1.63) | <0.02 |
| Sex | Maberley 2002 | 241 | 83 | Female | 1.17 (0.50–2.74) | >0.05 |
| Sex | Yun 2016 | 523 | 235 | Female | 1.02 (0.77–1.33) | 0.913 |
| Smoker | Anwar 2019 | 100 | 9 | Smoker | 1.01(0.11-8.96) | 0.991 |
| Smoker | Kumari 2019 | 759 | 93 | Smoker | 1.63 (1.02-2.62) | 0.041 |
| Smoker | Maberley 2002 | 241 | 83 | Smoker | 0.96 (0.39–2.35) | >0.05 |
| Smoker | Sardarinia 2022 | 1169 | 187 | Smoker | 1.87 (0.91–3.86) | 0.09 |
| Hypertension | Ahmed 2012 | 977 | 494 | Hypertension | 1.10 (0.91-1.32) | 0.309 |
| Hypertension | Anwar 2019 | 100 | 9 | Hypertension | 7.12(1.39–36.36) | 0.008 |
| Hypertension | Hammes 2015 | 64784 | 13034 | Hypertension | 1.15 (1.11-1.20) | <0.0001 |
| Hypertension | Kumari 2019 | 759 | 93 | Hypertension | 0.74 (0.48-1.13) | 0.164 |
| Hypertension | Liu 2015 | 622 | 52 | Hypertension | 1.49(1.12-1.73) | <0.01 |
| Hypertension | Looker 2003 | 280 | 75 | Hypertension | 0.64 (0.37–1.12) | >0.05 |
| Hypertension | Maberley 2002 | 241 | 83 | Hypertension | 1.08(0.45–2.63) | >0.05 |
| Hypertension | Salinero-Fort 2013 | 2405 | 194 | Hypertension | 0.95(0.70-1.29) | 0.745 |
| DM duration | Anwar 2019 | 100 | 9 | 11-20 (vs.0-10) years | 0.33 (0.03-3.43) | 0.037 |
| DM duration | Anwar 2019 | 100 | 9 | 21-30 (vs.0-10) years | 2.00 (0.18–22.06) | 0.037 |
| DM duration | Burgess 2017 | 135 | 40 | Increment by 1 year | 1.08(0.99-1.16) | 0.060 |
| DM duration | Kumari 2019 | 759 | 93 | Increment by 1 year | 1.02 (0.99-1.05) | 0.107 |
| DM duration | Liu 2015 | 622 | 52 | 5-10 (vs.<5) years | 1.86 (1.53-2.01) | <0.01 |
| DM duration | Liu 2015 | 622 | 52 | >10 (vs.<5) years | 2.28 (2.05-2.42) | <0.01 |
| DM duration | Looker 2003 | 280 | 75 | Increment by 1 year | 1.06 (1.01–1.11) | <0.05 |
| DM duration | Salinero-Fort 2013 | 2405 | 194 | 7-14 (vs.0-6) years | 1.22 (0.88–1.70) | 0.227 |
| DM duration | Salinero-Fort 2013 | 2405 | 194 | 15-22 (vs.0-6) years | 1.64 (1.05–2.57) | 0.029 |
| DM duration | Salinero-Fort 2013 | 2405 | 194 | >22 (vs.0-6) years | 2.00 (1.18–3.39) | 0.010 |
| DM duration | Salti 2009 | 500 | 175 | Increment by 10 years | 9.0 (4.0-20.0) | <0.001 |
| DM duration | Semeraro 2011 | 5034 | 569 | Increment by 1 year | 1.06 (1.05-1.08) | <0.001 |
| DM duration | Tung 2005 | 725 | 132 | Increment by 1 year | 1.09 (1.05-1.13) | <0.05 |
| DM duration | Wang 2022 | 1932 | 856 | Increment by 1 year | 1.17 (1.14-1.20) | <0.05 |
| DM duration | Yoshida 2001 | 787 | 132 | Increment by 1 year | 1.04(1.02-1.07) | 0.0018 |
| DM duration | Maberley 2002 | 241 | 83 | Increment by 10 years | 1.40(0.33-5.88) | >0.05 |
| DM duration | Yun 2016 | 523 | 235 | 5-10 (vs.<5) years | 1.50 (1.09–2.06) | 0.012 |
| DM duration | Yun 2016 | 523 | 235 | >10 (vs.<5) years | 1.98 (1.43–2.73) | <0.001 |
| Albuminuria | Hammes 2015 | 64784 | 13034 | Microalbuminuria | 1.16 (1.11-1.20) | <0.0001 |
| Albuminuria | Kumari 2019 | 759 | 93 | Macroalbuminuria | 1.15 (0.75-1.78) | 0.518 |
| Albuminuria | Looker 2003 | 280 | 75 | Macroalbuminuria | 2.86 (1.02–8.00) | <0.05 |
| Albuminuria | Salinero-Fort 2013 | 2405 | 194 | Microalbuminuria | 1.17(0.75-1.82) | 0.484 |
| Albuminuria | Salti 2009 | 500 | 175 | Macroalbuminuria | 2.6(1.14-5.96) | 0.023 |
| Albuminuria | Wang 2022 | 1932 | 856 | Microalbuminuria | 1.47 (1.15-1.87) | <0.05 |
| Albuminuria | Yun 2016 | 523 | 235 | Macroalbuminuria | 1.41 (1.04–1.93) | 0.029 |
| BMI | Hammes 2015 | 64784 | 13034 | >35 kg/m^2^ | 1.10 (1.05-1.16) | <0.01 |
| BMI | Kumari 2019 | 759 | 93 | Increment by 1 kg/m^2^ | 0.74 (0.6-0.93) | 0.009 |
| BMI | Looker 2003 | 280 | 75 | Increment by 1 kg/m^2^ | 1.00 (0.96–1.03) | >0.05 |
| BMI | Sardarinia 2022 | 1169 | 187 | 25-30 kg/m^2^ | 1.58 (0.67–3.72) | 0.29 |
| BMI | Sardarinia 2022 | 1169 | 187 | ≥30 kg/m^2^ | 1.87 (0.91–3.86) | 0.09 |
| BMI | Maberley 2002 | 241 | 83 | Increment by 5 kg/m^2^ | 0.64(0.04-1.00) | <0.05 |
| BMI | Yoshida 2001 | 787 | 132 | Increment by 1 kg/m^2^ | 0.98 (0.90–1.06) | 0.5857 |
| HbA1c | Ahmed 2012 | 977 | 494 | Increment by 1 %(11mmol/mol) | 0.52 (0.33-0.82) | 0.005 |
| HbA1c | Burgess 2017 | 135 | 40 | Increment by 1 %(11mmol/mol) | 1.23(1.03-1.48) | 0.020 |
| HbA1c | Chamard 2021 | 101321 | 16995 | Increment by 1 %(11mmol/mol) | 1.27 (1.22-1.31) | <0.0001 |
| HbA1c | Cheung 2021 | 498 | 96 | Increment by 1 %(11mmol/mol) | 1.28 (1.16-1.41) | <0.05 |
| HbA1c | Chiu 2021 | 792 | 611 | Increment by 1 %(11mmol/mol) | 1.07(1.02-1.12) | 0.009 |
| HbA1c | Hammes 2015 | 64784 | 13034 | (>8)% (>64 mmol/mol) (vs. <7%)(<53 mmol/mol) | 1.34 (1.29-1.39) | <0.0001 |
| HbA1c | Kumari 2019 | 759 | 93 | Increment by 1 %(11mmol/mol) | 1.41 (1.28-1.55) | <0.001 |
| HbA1c | Liu 2015 | 622 | 52 | (≥7)% (≥53 mmol/mol) (vs. <7%)(<53 mmol/mol) | 2.12(1.87-2.32) | <0.01 |
| HbA1c | Looker 2003 | 280 | 75 | Increment by 1 %(11mmol/mol) | 1.27 (1.16–1.41) | <0.05 |
| HbA1c | Salinero-Fort 2013 | 2405 | 194 | (7-8)% (53-64mmol/mol) (vs. <7%)(<53 mmol/mol) | 1.39 (1.01-1.92) | 0.044 |
| HbA1c | Salinero-Fort 2013 | 2405 | 194 | (>8)% (>64 mmol/mol) (vs. <7%)(<53 mmol/mol) | 1.90(1.30-2.77) | <0.001 |
| HbA1c | Salti 2009 | 500 | 175 | (>7)% (>53 mmol/mol) (vs. ≤7%)(≤53 mmol/mol) | 2.81(1.06-7.43) | 0.038 |
| HbA1c | Semeraro 2011 | 5034 | 569 | Increment by 1 %(11mmol/mol) | 1.16(1.09-1.25) | <0.001 |
| HbA1c | Tseng 2015 | 743 | 170 | Increment by 1 %(11mmol/mol) | 1.42 (1.18-1.72) | <0.001 |
| HbA1c | Tung 2005 | 725 | 132 | (7-8)% (53-64mmol/mol) (vs. <7%)(<53 mmol/mol) | 1.61(0.78-2.95) | >0.05 |
| HbA1c | Tung 2005 | 725 | 132 | (8-9)% (64-75mmol/mol) (vs. <7%)(<53 mmol/mol) | 3.09(1.67-5.70) | <0.05 |
| HbA1c | Tung 2005 | 725 | 132 | >9%(>75 mmol/mol) (vs. <7%)(<53 mmol/mol) | 3.29(1.97-5.49) | <0.05 |
| HbA1c | Wang 2022 | 1932 | 856 | Increment by 1 %(11mmol/mol) | 1.10 (1.02-1.18) | <0.05 |
| HbA1c | Yoshida 2001 | 787 | 132 | Increment by 1 %(11mmol/mol) | 1.47 (1.35-1.61) | 0.0001 |
| HbA1c | Yun 2016 | 523 | 235 | (7-9)% (53-75mmol/mol) (vs. <7%)(<53 mmol/mol) | 1.83 (1.08–3.09) | 0.025 |
| HbA1c | Yun 2016 | 523 | 235 | >9%(>75 mmol/mol) (vs. <7%)(<53 mmol/mol) | 4.32 (2.52–7.40) | <0.001 |
| SBP | Cheung 2021 | 498 | 96 | Increment by 10 mmHg | 1.14 (1.04-1.25) | <0.05 |
| SBP | Kumari 2019 | 759 | 93 | Increment by 1 mmHg | 1.00 (0.99-1.01) | 0.528 |
| SBP | Leske 2003 | 410 | 92 | Increment by 1 mmHg | 1.16 (1.03-1.31) | <0.05 |
| SBP | Salti 2009 | 500 | 175 | Increment by 10 mmHg | 1.27(1.0-1.56) | 0.037 |
| SBP | Semeraro 2011 | 5034 | 569 | Increment by 1 mmHg | 1.014(1.008-1.020) | <0.01 |
| SBP | Tseng 2015 | 743 | 170 | Increment by 1 mmHg | 1.03 (1.01-1.04) | 0.001 |
| SBP | Tung 2005 | 725 | 132 | ≥140 (vs.＜140) mmHg | 1.96(1.23-3.12) | <0.05 |
| SBP | Wang 2022 | 1932 | 856 | Increment by 1 mmHg | 1.02 (1.01-1.03) | <0.05 |
| SBP | Yoshida 2001 | 787 | 132 | Increment by 1 mmHg | 1.00(0.99-1.02) | 0.7001 |
| FBG | Looker 2003 | 280 | 75 | Increment by 1 mmol/L | 1.16 (1.10–1.22) | <0.05 |
| FBG | Sardarinia 2022 | 1169 | 187 | 7.22–10.0 (vs. <7.22) mmol/L | 3.51 (1.79–6.87) | <0.01 |
| FBG | Sardarinia 2022 | 1169 | 187 | ≥10.0 (vs. <7.22) mmol/L | 8.49 (4.36–16.50) | <0.01 |
| FBG | Wang 2022 | 1932 | 856 | Increment by 1 mmol/L | 1.07 (1.02-1.12) | <0.05 |
| LDL | Chiu 2021 | 792 | 611 | Increment by 1 mg/dL | 1.004(1.001-1.006) | 0.004 |
| LDL | Salinero-Fort 2013 | 2405 | 194 | 100-190 (vs.<100) mg/dL | 0.87 (0.65-1.16) | 0.332 |
| LDL | Salinero-Fort 2013 | 2405 | 194 | >190 (vs.<100) mg/dL | 7.91(3.39-18.47) | <0.001 |
| LDL | Yun 2016 | 523 | 235 | ≥2.6 (vs.<2.6) mg/ mmol/L | 1.09 (0.84–1.43) | 0.509 |
| TC | Ahmed 2012 | 977 | 494 | Increment by 1 mmol/L | 0.90 (0.74-1.08) | 0.276 |
| TC | Liu 2015 | 622 | 52 | >200 (vs.≤200) mg/dL | 1.54(1.34-1.72) | <0.01 |
| TC | Looker 2003 | 280 | 75 | Increment by 1 mmol/L | 0.95 (0.71–1.28) | >0.05 |
| TC | Tseng 2015 | 743 | 170 | Increment by 1 mmol/L | 1.01 (1.004-1.02) | 0.001 |
| TC | Maberley 2002 | 241 | 83 | >5.24 (vs.≤5.24) mmol/L | 2.38 (0.98–5.79) | >0.05 |
| TC | Yoshida 2001 | 787 | 132 | Increment by 1 mmol/L | 1.00(0.99-1.01) | 0.6347 |
| TG | Ahmed 2012 | 977 | 494 | Increment by 1 mmol/L | 1.06 (0.87-1.29) | 0.535 |
| TG | Burgess 2017 | 135 | 40 | Increment by 1 mmol/L | 0.66(0.35-1.24) | 0.195 |
| TG | Maberley 2002 | 241 | 83 | >2.55 (vs.≤2.55) mmol/L | 1.16(0.49–2.72) | >0.05 |
| TG | Tung 2005 | 725 | 132 | ≥200 (vs.<200) mg/dL | 1.60(1.01-2.54) | <0.05 |

Note: BMI, body mass index; HbA1c, Hemoglobin A1c; SBP, systolic blood pressure; FPG, fasting plasma glucose; LDL, Low density lipoprotein; TC, total cholesterol; TG, triglyceride;

**Supplementary Table 4 Publication bias**

| Risk factors | Begg’s test | | Egger’s test | |
| --- | --- | --- | --- | --- |
|  | Z | *P*r>\|z\| | t | *P* |
| Sex | 0.889 | 0.370 | 1.409 | 0.198 |
| Smoking | 1.042 | 0.294 | 4.213 | 0.141 |
| DM duration | 0.937 | 0.350 | 1.896 | 0.113 |
| Albuminuria | 0.601 | 0.543 | 4.159 | 0.151 |
| HbA1c | 0.310 | 0.743 | 1.932 | 0.132 |
| SBP | 0.728 | 0.469 | 1.214 | 0.339 |
| TG | 1.111 | 0.268 | 1.677 | 0.135 |

**Supplementary Table 5. Baseline characteristics of participants in validation cohort.**

| Variables | Total | Development of DR | | *P-*value |
| --- | --- | --- | --- | --- |
|  |  | Yes | No |  |
| N | 915 | 330 | 585 |  |
| Follow-up (months) | 32.0(22.0-40.0) | 32.5(19.0-40.0) | 32.0(23.0,40.0) | 0.595 |
| Age (years) | 53.6±11.4 | 52.7±10.8 | 54.1±11.6 | 0.06 |
| Male (%) | 574(62.7) | 225(68.2) | 349(59.7) | 0.01 |
| Smoker [n (%)] | 400(43.7) | 207(62.7) | 193(33.0) | <0.01 |
| Diabetes duration (years) | 8.0(3.0-13.0) | 9.0(5.0,14.1) | 7.0(2.0,12.5) | <0.01 |
| SBP (mmHg) | 130.0(120.0,140.0) | 135.0(129.3,150.0) | 130.0(120.0,140.0) | <0.01 |
| HbA1c (%)  [mmol/mol] | 8.4(7.2,9.8)  [68.0(55.0,84.0)] | 9.1(8.1,10.3)  [76.0(65.0,89.0)] | 8.0(6.9,9.4)  [64.0(52.0,79.0)] | <0.01 |
| TG (mmol/L) | 1.74(1.24,2.69) | 2.13(1.35,3.38) | 1.61(1.20,2.40) | <0.01 |
| Albuminuria  [n (%)] |  |  |  | <0.01 |
| No | 661(72.2) | 180(54.5) | 481(82.2) |  |
| Microalbuminuria | 221(24.2) | 126(38.2) | 95(16.2) |  |
| Macroalbuminuria | 33(3.6) | 24(7.3) | 9(1.5) |  |
| OAD [n (%)] | 872(95.3) | 315(95.5) | 557(95.2) | 0.869 |
| Insulin [n (%)] | 488(53.3) | 182(55.2) | 306(52.3) | 0.408 |

**Supplementary Table 6: The sensitivity, specificity and Youden index of different cutoff risk scores in DR risk prediction model.**

| Cut-off  value | Sensitivity | Specificity | Youden  index |
| --- | --- | --- | --- |
| -1.00 | 1.000 | 1.000 | 0.000 |
| 0.50 | 1.000 | 0.998 | 0.002 |
| 1.50 | 1.000 | 0.997 | 0.003 |
| 2.00 | 1.000 | 0.986 | 0.014 |
| 2.50 | 1.000 | 0.959 | 0.041 |
| 3.00 | 1.000 | 0.956 | 0.044 |
| 3.50 | 1.000 | 0.952 | 0.048 |
| 4.00 | 1.000 | 0.916 | 0.084 |
| 4.50 | 0.997 | 0.906 | 0.091 |
| 5.00 | 0.994 | 0.891 | 0.103 |
| 5.50 | 0.988 | 0.867 | 0.121 |
| 6.00 | 0.970 | 0.838 | 0.132 |
| 6.50 | 0.967 | 0.805 | 0.162 |
| 7.00 | 0.961 | 0.778 | 0.183 |
| 7.50 | 0.948 | 0.756 | 0.193 |
| 8.00 | 0.948 | 0.747 | 0.201 |
| 8.50 | 0.936 | 0.706 | 0.230 |
| 9.00 | 0.924 | 0.679 | 0.246 |
| 9.50 | 0.921 | 0.662 | 0.260 |
| 10.00 | 0.900 | 0.631 | 0.269 |
| 10.50 | 0.888 | 0.593 | 0.295 |
| 11.00 | 0.885 | 0.566 | 0.319 |
| 11.50 | 0.861 | 0.549 | 0.312 |
| 12.00 | 0.855 | 0.530 | 0.325 |
| 12.50 | 0.833 | 0.499 | 0.334 |
| 13.00 | 0.824 | 0.441 | 0.383 |
| 13.50 | 0.812 | 0.415 | 0.397 |
| 14.00 | 0.788 | 0.378 | 0.410 |
| 14.50 | 0.770 | 0.349 | 0.421 |
| 15.00 | 0.742 | 0.318 | 0.424 |
| 15.50 | 0.730 | 0.280 | 0.450 |
| 16.00^*^ | 0.715 | 0.255 | 0.460 |
| 16.50 | 0.688 | 0.238 | 0.450 |
| 17.00 | 0.645 | 0.214 | 0.432 |
| 17.50 | 0.609 | 0.198 | 0.411 |
| 18.00 | 0.567 | 0.178 | 0.389 |
| 18.50 | 0.521 | 0.162 | 0.359 |
| 19.00 | 0.491 | 0.152 | 0.339 |
| 19.50 | 0.430 | 0.138 | 0.292 |
| 20.00 | 0.409 | 0.118 | 0.291 |
| 20.50 | 0.385 | 0.099 | 0.286 |
| 21.00 | 0.336 | 0.079 | 0.258 |
| 21.50 | 0.312 | 0.068 | 0.244 |
| 22.00 | 0.270 | 0.053 | 0.217 |
| 22.50 | 0.248 | 0.046 | 0.202 |
| 23.00 | 0.221 | 0.039 | 0.182 |
| 23.50 | 0.197 | 0.038 | 0.159 |
| 24.00 | 0.170 | 0.032 | 0.137 |
| 24.50 | 0.152 | 0.021 | 0.131 |
| 25.00 | 0.121 | 0.019 | 0.102 |
| 25.50 | 0.100 | 0.015 | 0.085 |
| 26.00 | 0.082 | 0.014 | 0.068 |
| 26.50 | 0.076 | 0.009 | 0.067 |
| 27.00 | 0.070 | 0.009 | 0.061 |
| 27.50 | 0.061 | 0.007 | 0.054 |
| 28.00 | 0.039 | 0.003 | 0.036 |
| 28.50 | 0.033 | 0.000 | 0.033 |
| 29.00 | 0.027 | 0.000 | 0.027 |
| 29.75 | 0.021 | 0.000 | 0.021 |
| 30.50 | 0.009 | 0.000 | 0.009 |
| 32.75 | 0.006 | 0.000 | 0.006 |
| 35.00 | 0.000 | 0.000 | 0.000 |

* Optimal cutoff point

**Supplementary Figure1:** **RR (95% CI) and** **results of heterogeneity test of the risk factors for DR.**


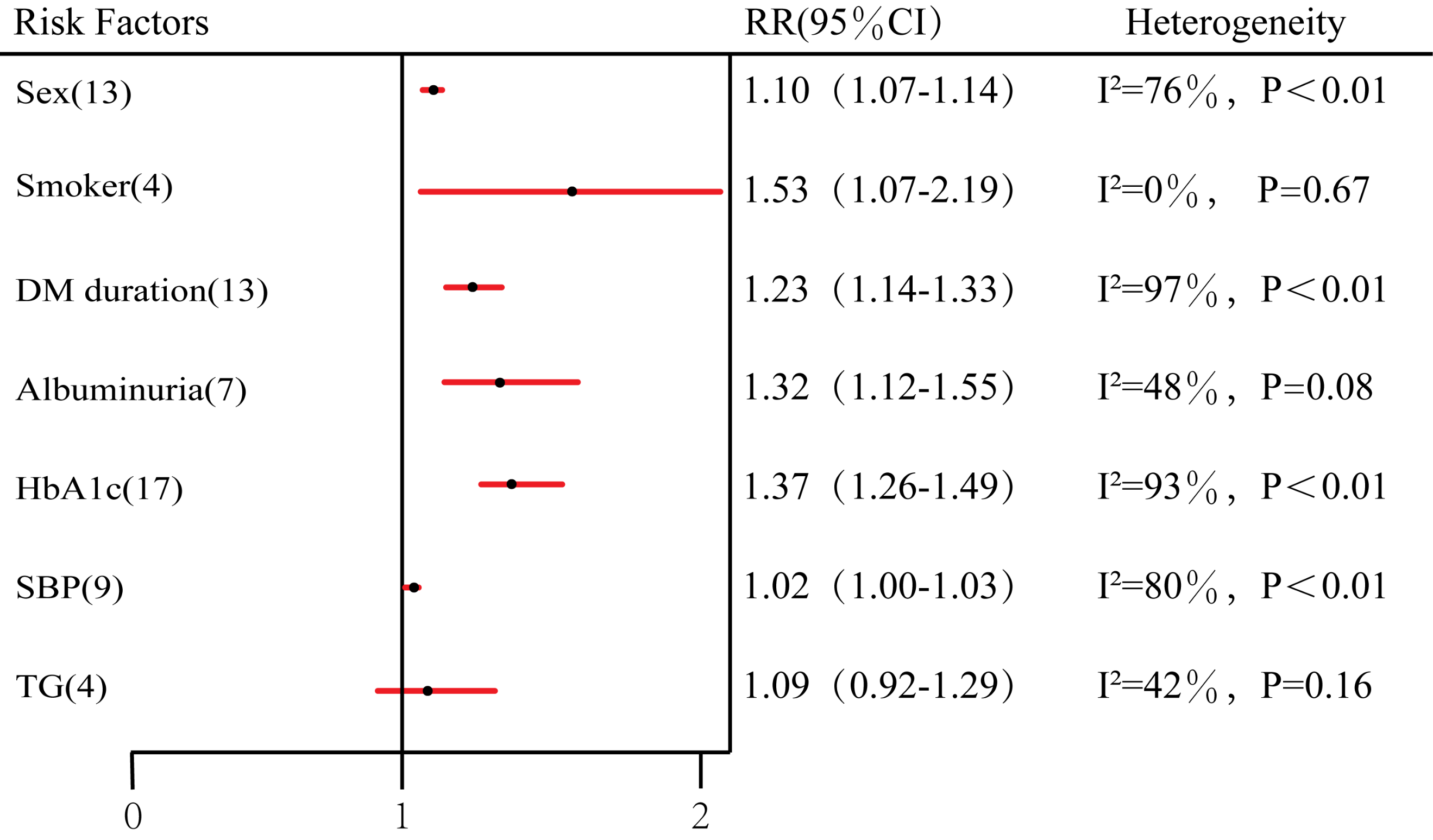


**Supplementary Figure2:** **Subgroup or sensitivity analyses of the risk factors for DR.**


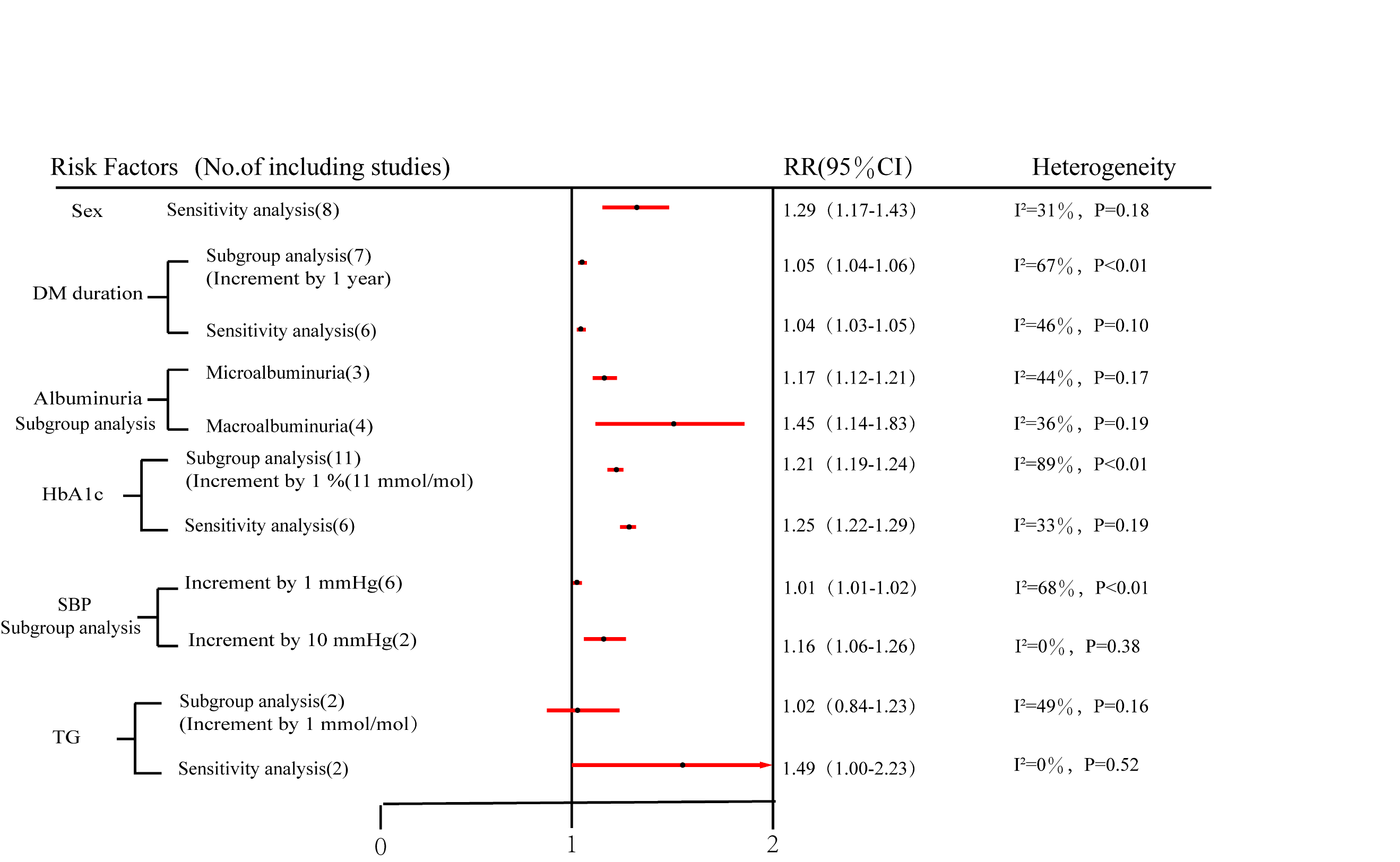


**Supplementary Figure 3. Sex**

**
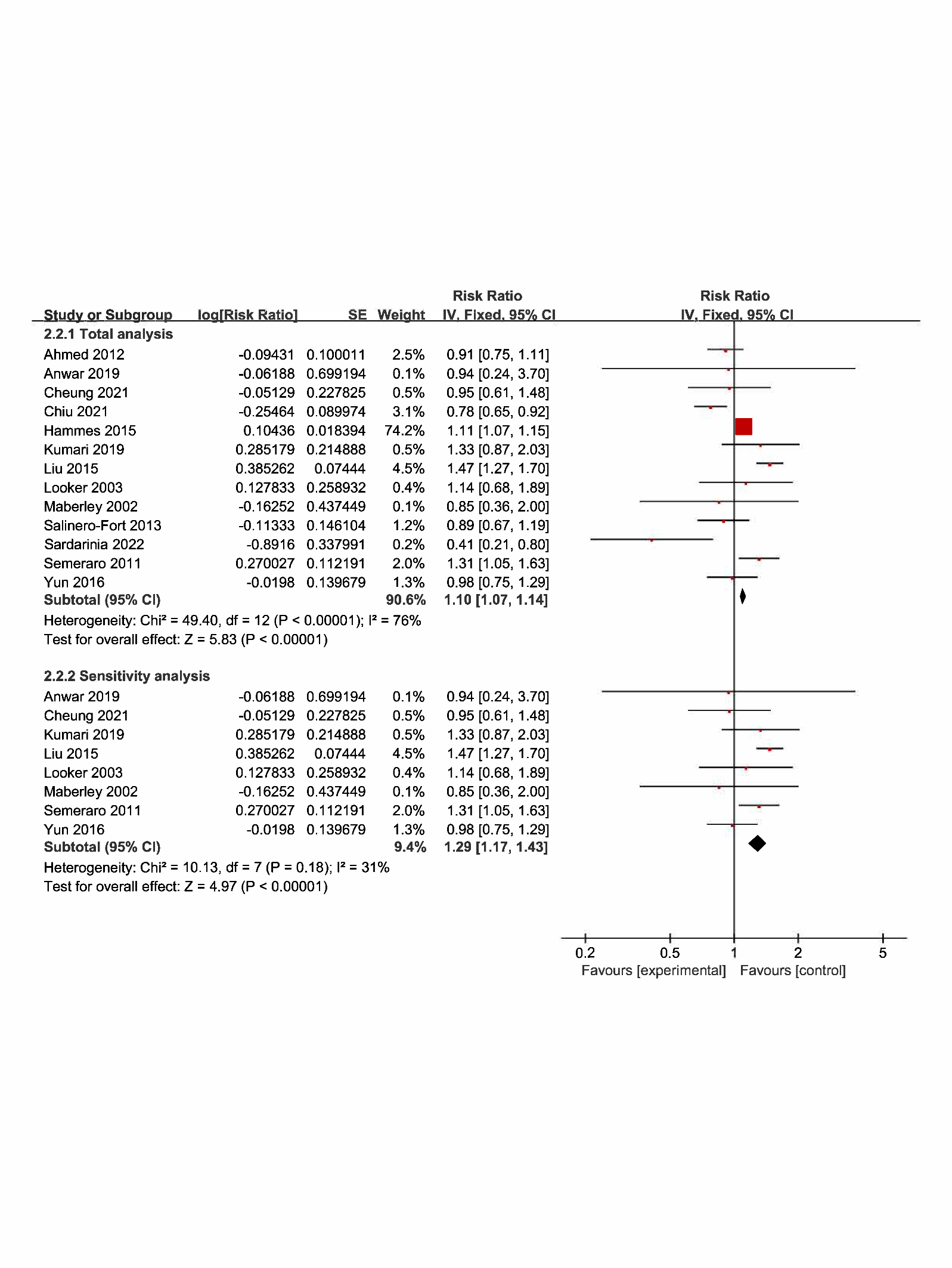
**

Supplementary Figure 3. Association of sex with DR

**Supplementary Figure 4. Smoking**

**
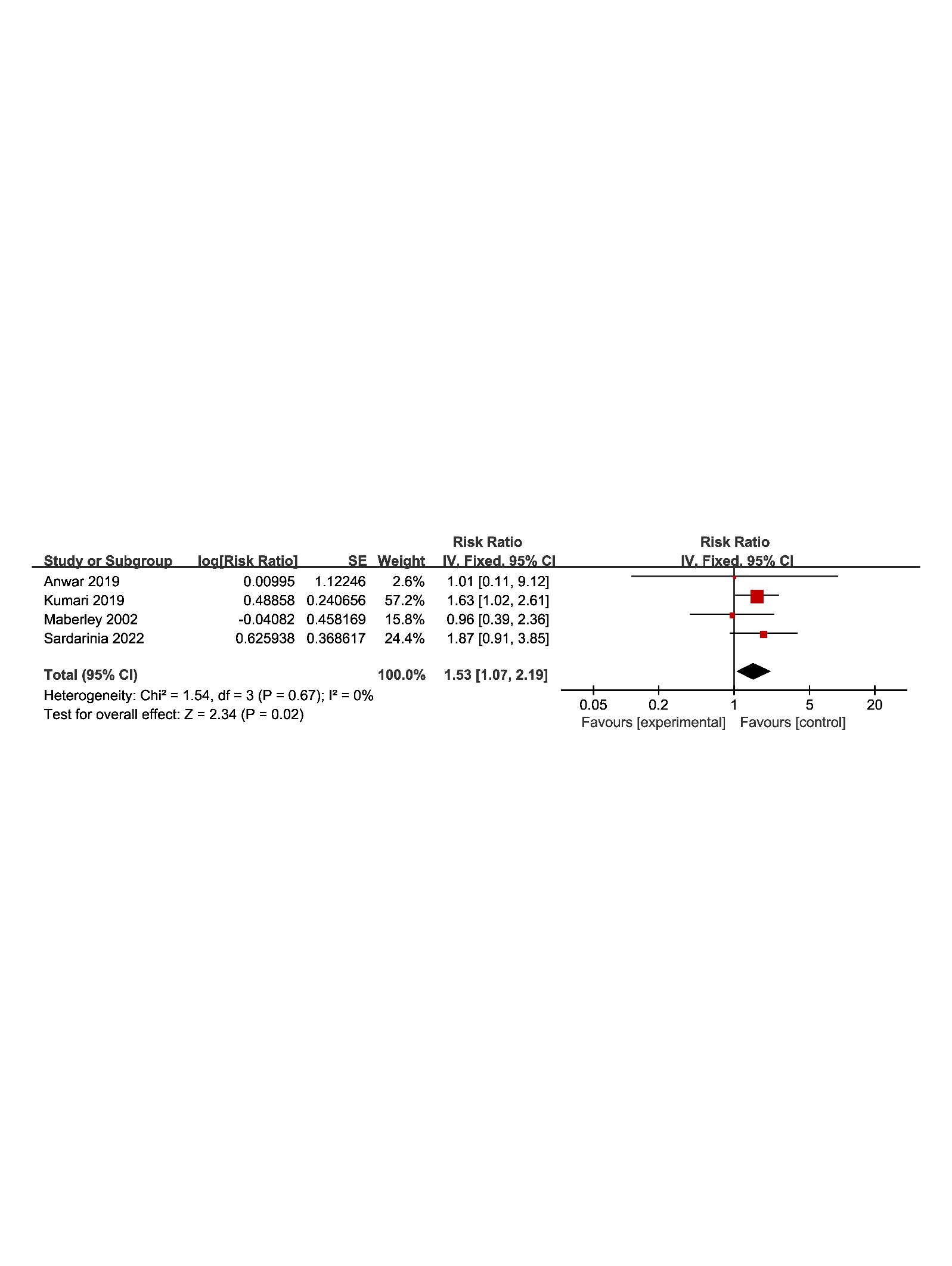
**

Supplementary Figure 4. Association of smoking with DR

**Supplementary Figure 5. DM duration**


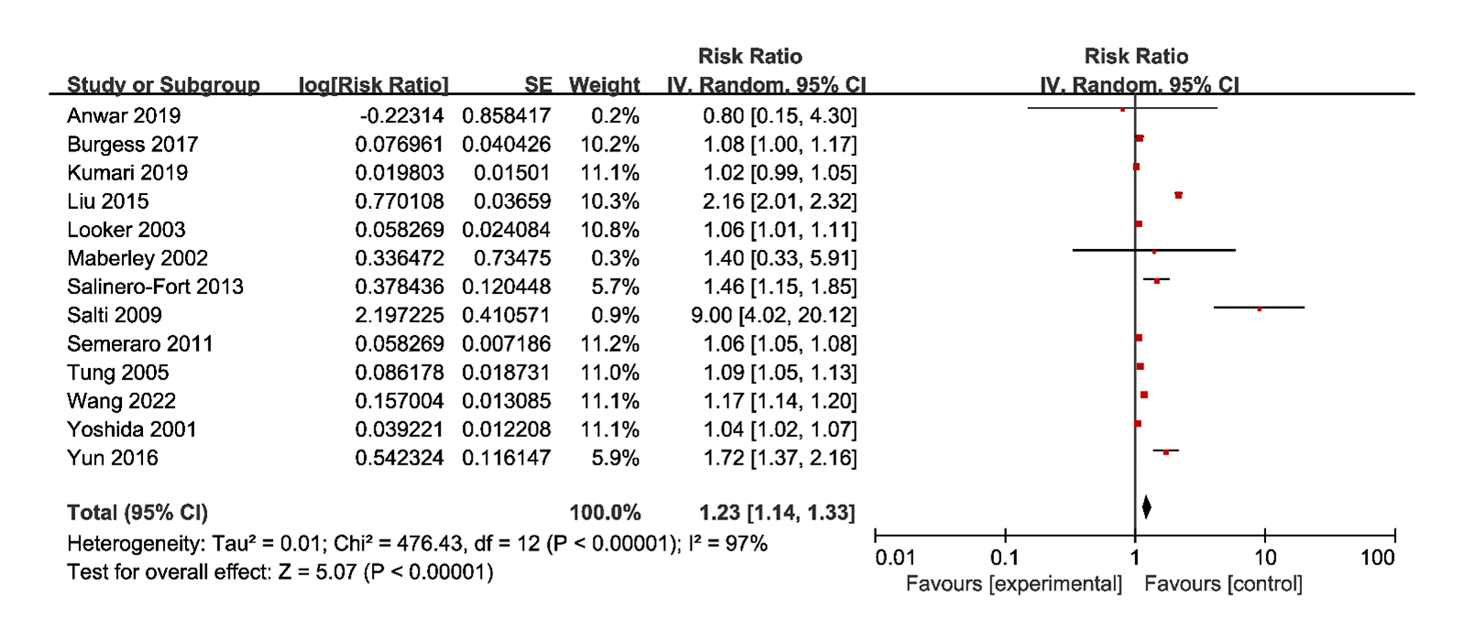


B

A


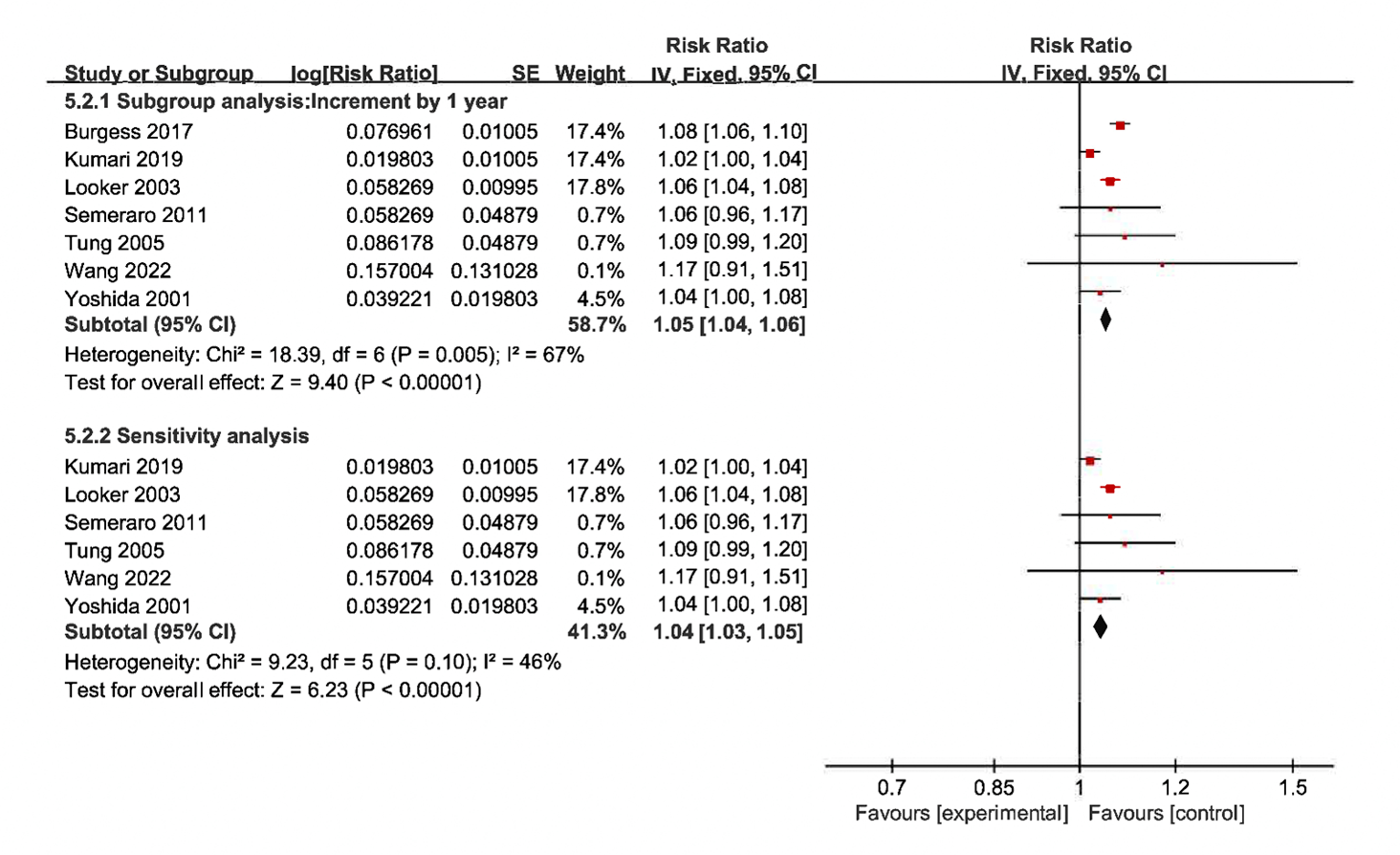


Figure 5- A. Association of DM duration with DR; B. Sensitivity analysis and subgroup analysis of association of DM duration with DR.

**Supplementary Figure 6. Albuminuria**


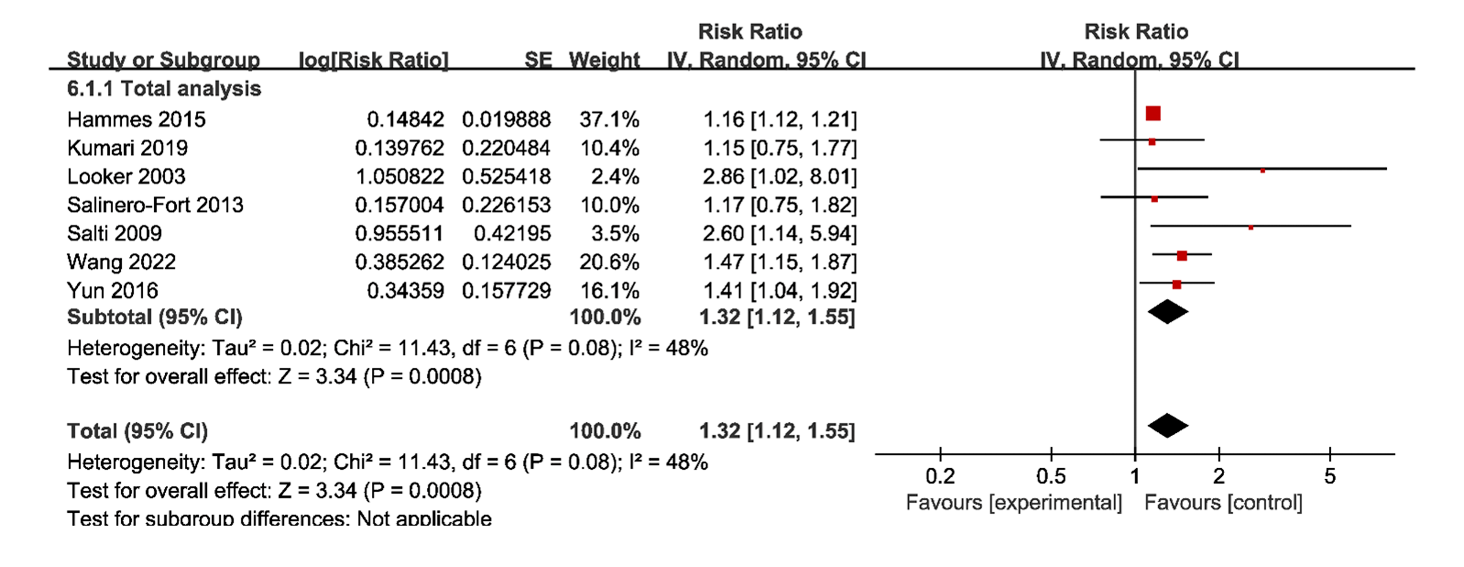


A

B


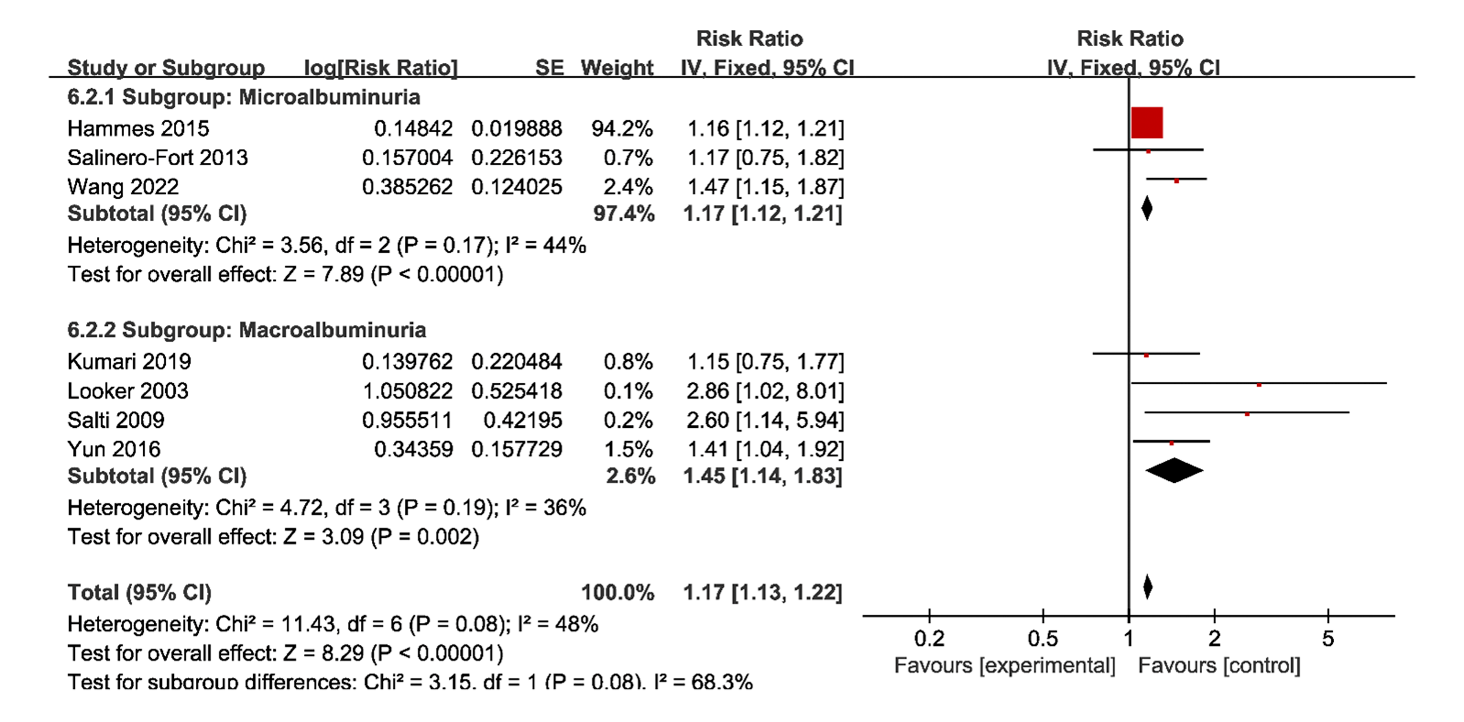


Figure 6- A. Association of Albuminuria with DR; B. Subgroup analysis of association of Albuminuria with DR.

**Supplementary Figure 7. HbA1c**


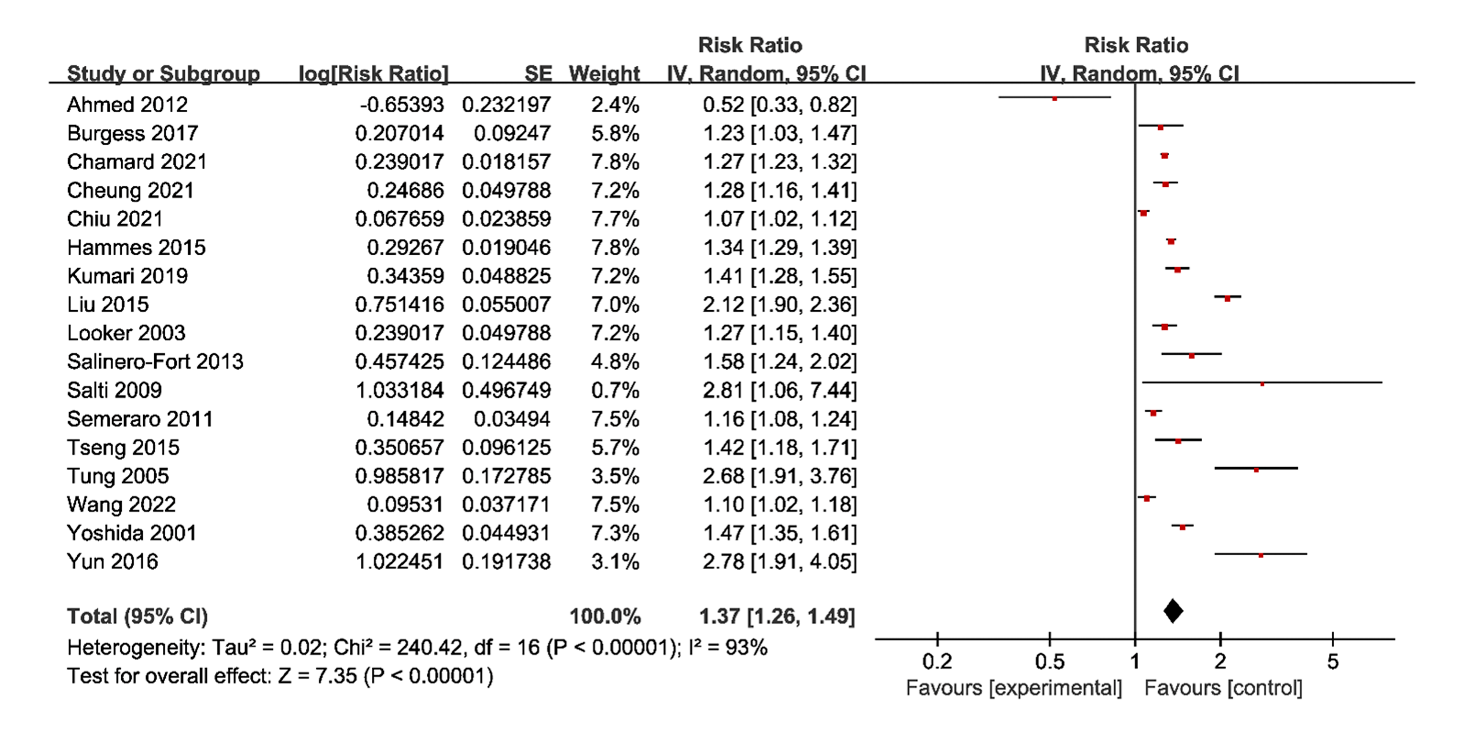


B

A


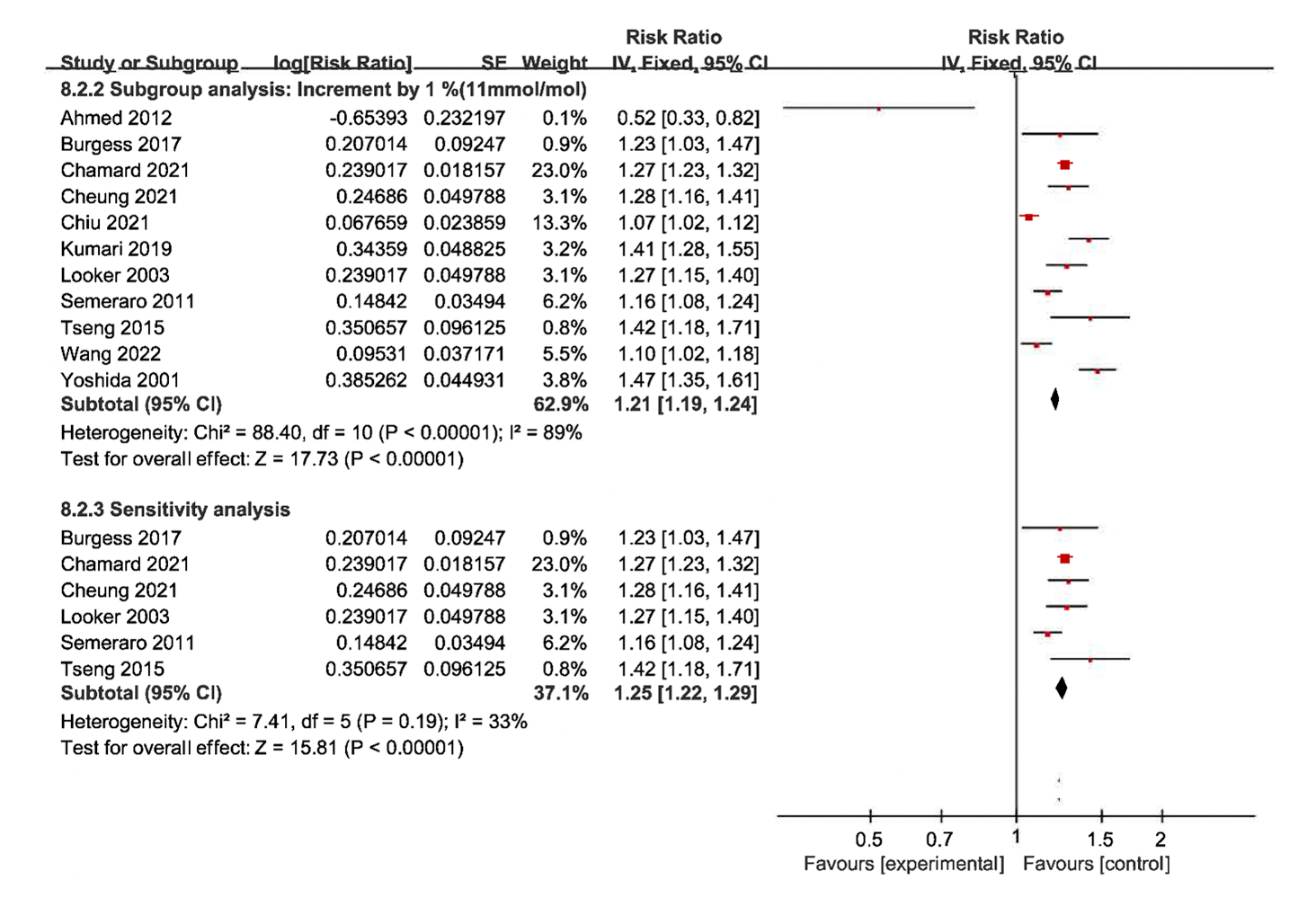


Figure 7- A. Association of HbA1c with DR; B. Sensitivity analysis and subgroup analysis of association of HbA1c with DR.

**Supplementary Figure 8. SBP**


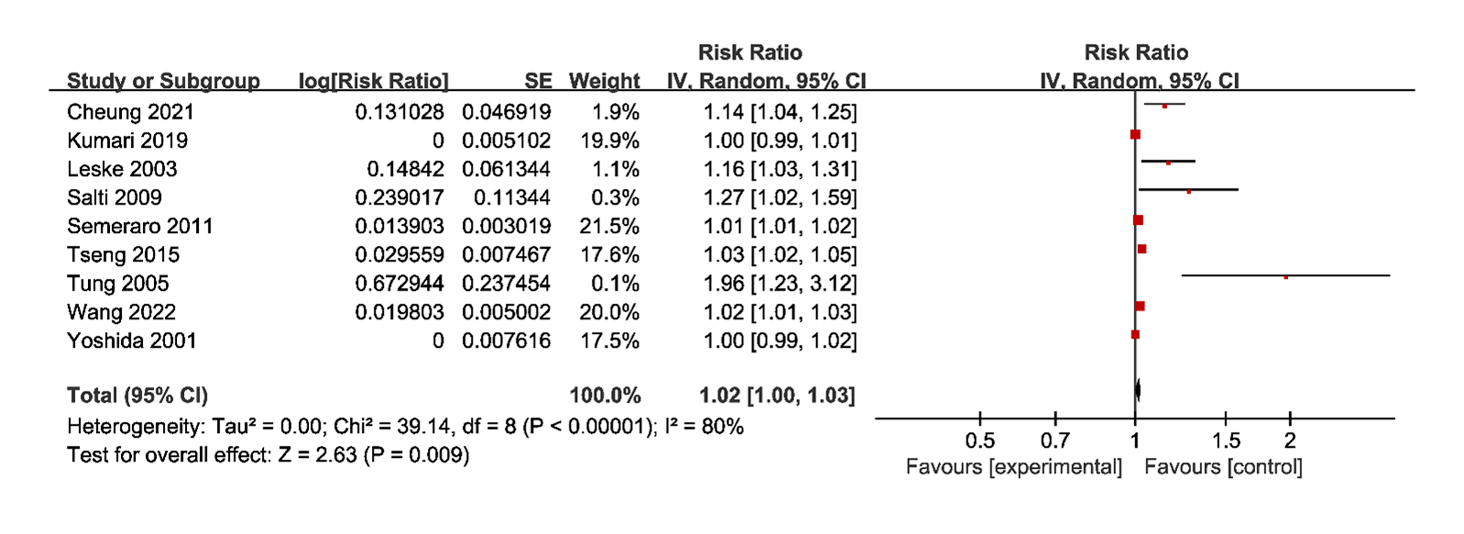


B

A


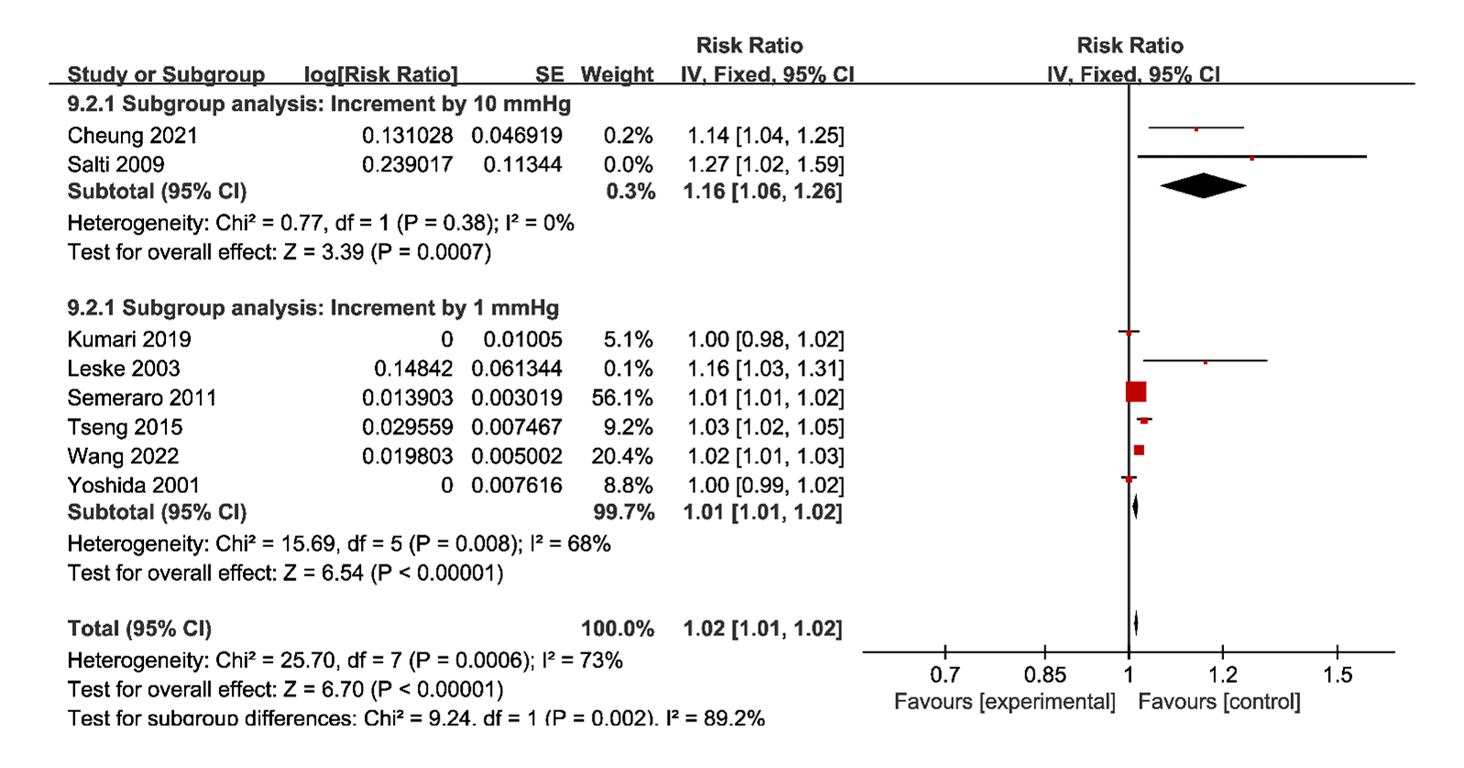


Figure 8- A. Association of SBP with DR; B. Subgroup analysis of association of SBP with DR.

**Supplementary Figure 9. TG**


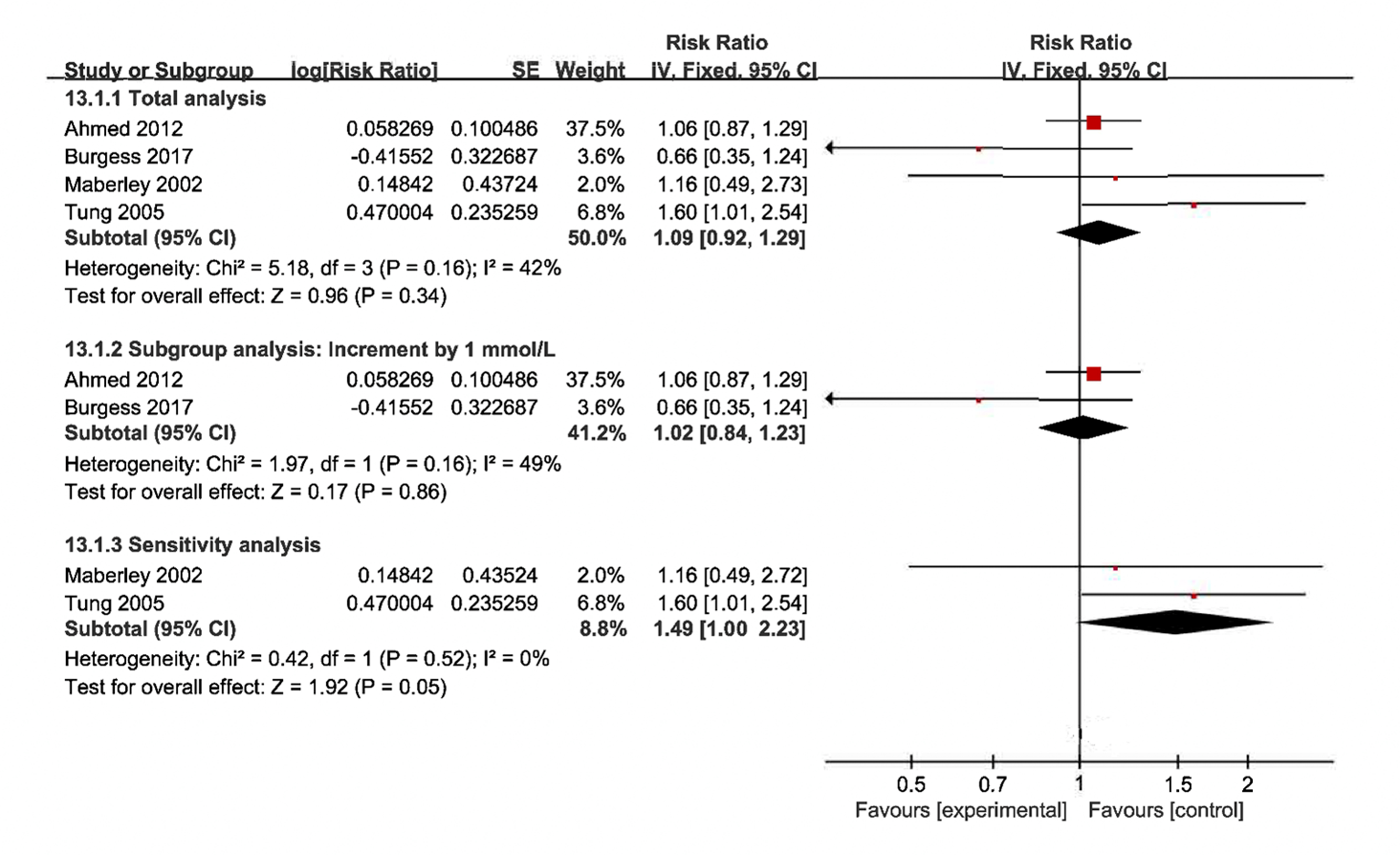


Figure 9. Sensitivity analysis and subgroup analysis of association of TG with DR.

Reference

[1] Shamseer L, Moher D, Clarke M, et al. Preferred reporting items for systematic review and meta-analysis protocols (PRISMA-P) 2015: elaboration and explanation [J]. Bmj, 2015, 350: g7647.

[2] Stroup D F, Berlin J A, Morton S C, et al. Meta-analysis of observational studies in epidemiology: a proposal for reporting. Meta-analysis Of Observational Studies in Epidemiology (MOOSE) group [J]. Jama, 2000, 283(15): 2008-2012.

[3] Alberti K G, Zimmet P Z. Definition, diagnosis and classification of diabetes mellitus and its complications. Part 1: diagnosis and classification of diabetes mellitus provisional report of a WHO consultation [J]. Diabet Med, 1998, 15(7): 539-553.

[4] 2. Classification and Diagnosis of Diabetes: Standards of Medical Care in Diabetes-2019 [J]. Diabetes Care, 2019, 42(Suppl 1): S13-s28.

[5] Yun J S, Lim T S, Cha S A, et al. Clinical course and risk factors of diabetic retinopathy in patients with type 2 diabetes mellitus in korea [J]. Diabetes and Metabolism Journal, 2016, 40(6): 482-493.

[6] Tung T H, Chen S J, Liu J H, et al. A community-based follow-up study on diabetic retinopathy among type 2 diabetics in Kinmen [J]. Eur J Epidemiol, 2005, 20(4): 317-323.

[7] Anwar S B, Asif N, Naqvi S a H, et al. Evaluation of multiple risk factors involved in the development of diabetic retinopathy [J]. Pakistan Journal of Medical Sciences, 2019, 35(1): 156-160.

[8] Cikamatana L, Mitchell P, Rochtchina E, et al. Five-year incidence and progression of diabetic retinopathy in a defined older population: The Blue Mountains Eye Study [J]. Eye, 2007, 21(4): 465-471.

[9] Manaviat M R, Rashidi M, Afkhami-Ardekani M. Four years incidence of diabetic retinopathy and effective factors on its progression in type II diabetes [J]. Eur J Ophthalmol, 2008, 18(4): 572-577.

[10] Salinero-Fort M, San Andrés-Rebollo F J, De Burgos-Lunar C, et al. Four-year incidence of diabetic retinopathy in a Spanish cohort: the MADIABETES study [J]. PLoS One, 2013, 8(10): e76417.

[11] Jones C D, Greenwood R H, Misra A, et al. Incidence and progression of diabetic retinopathy during 17 years of a population-based screening program in England [J]. Diabetes Care, 2012, 35(3): 592-596.

[12] Cheung N, Chee M L, Klein R, et al. Incidence and progression of diabetic retinopathy in a multi-ethnic US cohort: The Multi-Ethnic Study of Atherosclerosis [J]. British Journal of Ophthalmology, 2021.

[13] Tudor S M, Hamman R F, Baron A, et al. Incidence and progression of diabetic retinopathy in Hispanics and non-Hispanic whites with type 2 diabetes. San Luis Valley Diabetes Study, Colorado [J]. Diabetes Care, 1998, 21(1): 53-61.

[14] Burgess P I, Harding S P, García-Fiñana M, et al. Incidence and progression of diabetic retinopathy in Sub-Saharan Africa: A five year cohort study [J]. PLoS ONE, 2017, 12(8).

[15] Sardarinia M, Asgari S, Hizomi Arani R, et al. Incidence and risk factors of severe non-proliferative/proliferative diabetic retinopathy: More than a decade follow up in the Tehran Lipids and Glucose Study [J]. J Diabetes Investig, 2022, 13(2): 317-327.

[16] Liu L, Wu J, Yue S, et al. Incidence density and risk factors of diabetic retinopathy within type 2 diabetes: A five-year cohort study in China (Report 1) [J]. International Journal of Environmental Research and Public Health, 2015, 12(7): 7899-7909.

[17] Ahmed K R, Karim M N, Bhowmik B, et al. Incidence of diabetic retinopathy in Bangladesh: A 15-year follow-up study [J]. Journal of Diabetes, 2012, 4(4): 386-391.

[18] Leske M C, Wu S Y, Hennis A, et al. Incidence of diabetic retinopathy in the Barbados Eye Studies [J]. Ophthalmology, 2003, 110(5): 941-947.

[19] Looker H C, Krakoff J, Knowler W C, et al. Longitudinal studies of incidence and progression of diabetic retinopathy assessed by retinal photography in Pima Indians [J]. Diabetes Care, 2003, 26(2): 320-326.

[20] Semeraro F, Parrinello G, Cancarini A, et al. Predicting the risk of diabetic retinopathy in type 2 diabetic patients [J]. J Diabetes Complications, 2011, 25(5): 292-297.

[21] Salti H I, Nasrallah M P, Taleb N M, et al. Prevalence and determinants of retinopathy in a cohort of Lebanese type II diabetic patients [J]. Can J Ophthalmol, 2009, 44(3): 308-313.

[22] Wang Y, Lin Z, Zhai G, et al. Prevalence of and Risk Factors for Diabetic Retinopathy and Diabetic Macular Edema in Patients with Early- and Late-Onset Diabetes Mellitus [J]. Ophthalmic Research, 2022, 65(3): 293-299.

[23] Chiu T T, Tsai T L, Su M Y, et al. The related risk factors of diabetic retinopathy in elderly patients with type 2 diabetes mellitus: A hospital-based cohort study in Taiwan [J]. International Journal of Environmental Research and Public Health, 2021, 18(1): 1-9.

[24] Tseng S T, Chou S T, Low B H, et al. Risk factors associated with diabetic retinopathy onset and progression in diabetes patients: A Taiwanese cohort study [J]. International Journal of Clinical and Experimental Medicine, 2015, 8(11): 21507-21515.

[25] Araki A, Ito H, Hattori A, et al. Risk factors for development of retinopathy in elderly Japanese patients with diabetes mellitus [J]. Diabetes Care, 1993, 16(8): 1184-1186.

[26] Maberley D a L, King W, Cruess A F, et al. Risk factors for diabetic retinopathy in the Cree of James Bay [J]. Ophthalmic Epidemiology, 2002, 9(3): 153-167.

[27] Hammes H P, Welp R, Kempe H P, et al. Risk factors for retinopathy and DME in type 2 diabetes-results from the German/Austrian DPV database [J]. PLoS ONE, 2015, 10(7).

[28] Yoshida Y, Hagura R, Hara Y, et al. Risk factors for the development of diabetic retinopathy in Japanese type 2 diabetic patients [J]. Diabetes Res Clin Pract, 2001, 51(3): 195-203.

[29] Kumari N, Bhargava M, Nguyen D Q, et al. Six-year incidence and progression of diabetic retinopathy in Indian adults: the Singapore Indian Eye study [J]. Br J Ophthalmol, 2019, 103(12): 1732-1739.

[30] Chamard C, Daien V, Erginay A, et al. Ten-year incidence and assessment of safe screening intervals for diabetic retinopathy: The OPHDIAT study [J]. British Journal of Ophthalmology, 2021, 105(3): 432-439.
